# Nonbinary 2D Distribution Tool Maps Autonomic Nerve Fiber Clustering in Lumbosacral Ventral Roots of Rhesus Macaques

**DOI:** 10.1523/ENEURO.0009-23.2024

**Published:** 2024-04-11

**Authors:** Petra M. Bartmeyer, Natalia P. Biscola, Leif A. Havton

**Affiliations:** ^1^Departments of Neurology, Icahn School of Medicine at Mount Sinai, New York, New York 10029; ^2^Neuroscience, Icahn School of Medicine at Mount Sinai, New York, New York 10029; ^3^James J. Peters Veterans Affairs Medical Center, Bronx, New York 10468

**Keywords:** cluster analysis, heterogeneity, morphologic mapping, neuromodulation, rhesus macaque

## Abstract

Neuromodulation of the peripheral nervous system (PNS) by electrical stimulation may augment autonomic function after injury or in neurodegenerative disorders. Nerve fiber size, myelination, and distance between individual fibers and the stimulation electrode may influence response thresholds to electrical stimulation. However, information on the spatial distribution of nerve fibers within the PNS is sparse. We developed a new two-dimensional (2D) morphological mapping tool to assess spatial heterogeneity and clustering of nerve fibers. The L6-S3 ventral roots (VRs) in rhesus macaques were used as a model system to map preganglionic parasympathetic, γ-motor, and α-motor fibers. Random and ground truth distributions of nerve fiber centroids were determined for each VR by light microscopy. The proposed tool allows for nonbinary determinations of fiber heterogeneity by defining the minimum distance between nerve fibers for cluster inclusion and comparisons with random fiber distributions for each VR. There was extensive variability in the relative composition of nerve fiber types and degree of 2D fiber heterogeneity between different L6-S3 VR levels within and across different animals. There was a positive correlation between the proportion of autonomic fibers and the degree of nerve fiber clustering. Nerve fiber cluster heterogeneity between VRs may contribute to varied functional outcomes from neuromodulation.

## Significance Statement

Neuromodulation of the peripheral nervous system (PNS) by electrical stimulation may augment autonomic function after injury or in neurodegenerative disorders. Nerve fiber size, myelination, and distance between individual fibers may influence response thresholds to electrical stimulation. However, information on spatial distribution of nerve fibers within the PNS is sparse. We developed a two-dimensional morphological mapping tool to assess spatial heterogeneity and clustering of nerve fibers in lumbosacral ventral roots of rhesus macaques. The tool allows for nonbinary determinations of fiber heterogeneity by defining the minimum distance between nerve fibers for cluster inclusion and from comparisons with random fiber distributions for each ventral root. Nerve fiber cluster heterogeneity between ventral roots may contribute to varied functional outcomes from neuromodulation.

## Introduction

The peripheral nervous system (PNS) represents an attractive therapeutic target for neuromodulation of autonomic functions, including electrical stimulation of select nerve roots and peripheral nerves to reverse conditions. For instance, tibial nerve, sacral anterior root, or sacral nerve stimulation may ameliorate neurogenic bladder syndromes (Brindley, 1994; Peters et al., 2009; Sievert et al., 2010; Martens et al., 2011; Janssen et al., 2017). Additional and emerging neuromodulation strategies, including epidural and transcutaneous spinal cord stimulation, also include electrical activation of nerve roots to evoke micturition reflexes and augment bladder emptying after spinal cord injury in animal models and clinical studies (Abud et al., 2015; Gad et al., 2018; Herrity et al., 2018, 2022). However, therapeutic responses to electrical stimulation of nervous structures vary between subjects, and underlying mechanisms of action are incompletely understood.

The autonomic nervous system presents with unique organizational features (Shields, 1993; Furness, 2006; Jänig, 2006), and they will need to be taken into consideration for the development of refined or new neuromodulation strategies to augment visceral organ function in clinical conditions. For instance, autonomic nerve fibers may extend in the presence of a varied number of somatic motor and sensory fibers in nerve roots and peripheral nerves (Goldstein, 2001; Smith, 2006). The likelihood of individual nerve fiber activation by electrical stimulation is influenced by, for instance, nerve fascicular size and organization, perineurium thickness, and distance from the stimulating electrode (Grinberg et al., 2008; Zellmer et al., 2018; Musselman et al., 2021). Conduction and recruitment properties of nerve fibers to electrical stimulation are also affected by their axonal caliber and myelination (Erlanger and Gasser, 1924; Waxman, 1980; de Moraes et al., 2017). Quantitative studies of nerve roots and peripheral nerves have recently been able to incorporate size correction factors and provide fiber size determinations closer to ground truth data (Bartmeyer et al., 2021; Havton et al., 2021). However, a practical approach to determine the two-dimensional (2D) distribution of nerve fibers in the PNS has been in short supply and is much needed to develop the next generation of refined stimulation strategies for neuromodulation of the PNS (Eiber et al., 2021; Musselman et al., 2021).

To address this critical need, we have developed a new 2D mapping tool for the identification of nerve fiber clusters in the PNS. The tool is size-neutral and nonbinary, so that clustering of nerve fibers may be quantified independently from nerve root or peripheral nerve cross-sectional area, perimeter, shape, or the number of nerve fibers included in the analysis. As a model system, we used the L6-S3 ventral roots (VRs) in nonhuman primates (NHPs) to show feasibility and utility for the new analytical tool. Both autonomic and motor fibers exit the conus medullaris of the spinal cord and extend into lumbosacral VRs in rhesus macaques (Sherrington, 1892), but individual lumbosacral VRs vary extensively with regard to size, number of myelinated fibers, and relative proportions of autonomic and motor fibers (Ohlsson et al., 2013; Bartmeyer et al., 2021). We quantified heterogeneity of nerve fiber distribution in VRs and mapped nerve fiber clustering of both whole and subpopulations of nerve fibers. An association between the presence of autonomic fibers and degree of nerve fiber clustering in lumbosacral VRs in rhesus macaques was also determined.

## Materials and Methods

The ventral root (VR) tissues subject to morphological analysis in the present studies represent archival material and were procured in conjunction with prior investigations on the effects of lumbosacral ventral root avulsion injury in female rhesus macaques (Chang et al., 2018; Nieto et al., 2019; Bartmeyer et al., 2021). All spine surgical procedures and procurement of VR tissues were performed at the California National Primate Research Center at UC Davis, an academic institution accredited by the Association for Assessment and Accreditation of Laboratory Animal Care (AAALAC). All procedures were approved by the Institutional Animal Care and Use Committee (IACUC) at UC Davis, were performed in compliance with the Guide for the Care and Use of Laboratory Animals (Institute for Laboratory Animal Research), and followed ARRIVE 2.0 guidelines for the care and use of laboratory animals (Percie du Sert et al., 2020).

### Animal procedures and VR processing

VRs from a total of six female rhesus macaques were included in the studies. The animals were 9.0 ± 0.7 years old and with a weight of 8.1 ± 0.8 kg. Spine surgery, identification of VRs, and root procurement were performed according to our established protocols and procedures (Ohlsson et al., 2013; Chang et al., 2018; Nieto et al., 2019; Bartmeyer et al., 2021). In short, spine radiographs and lumbar magnetic resonance imaging (MRI) were first obtained as part of the surgical planning for each animal to determine spine anatomy, including possible presence of thoracolumbar spine variants, and rostro-caudal positioning of the conus medullaris within the spinal canal (Ohlsson et al., 2017). A surgical plane of anesthesia was provided by 1–2% isoflurane via endotracheal tube and fentanyl by intravenous administration. Following a midline lumbar skin incision and a left-sided laminectomy extending from the caudal aspect of the L1 vertebra to the rostral aspect of the L3 vertebra and opening of the dura mater, the left-sided L6-S3 VRs were identified and avulsed from the surface of the spinal cord by gentle traction with the use of a pair of fine forceps. An approximately 5 mm long segment was excised from each of the avulsed VRs and placed in a solution of 2% paraformaldehyde + 2.5% glutaraldehyde in phosphate buffered saline at pH 7.4. A continuous 6-0 Ethilon suture was used to close the dura mater, and 4-0 Vicryl sutures were used to close paraspinous muscles and skin in layers. All animals recovered well from the surgical procedure, received scheduled and as needed analgesic agents for pain control, and were monitored closely clinically during the post-operative recovery period. The procured VRs were immersion-fixed for 24–48 h, rinsed in phosphate buffer, treated in 1% osmium tetroxide (OsO_4_), dehydrated in ethanol, infiltrated with 50% propylene oxide + 50% Epon plastic resin, and embedded in 100% Epon (Bartmeyer et al., 2021). Transverse sections of the VRs were next cut at approximately 0.5 µm thickness, mounted onto glass slides, stained with a 1% toluidine blue solution, and cover-slipped for light microscopy (LM).

### Microscopy and nerve fiber segmentation

A Nikon E600 light microscope, equipped with a DS-Fi3 camera and Nikon NIS-Elements software, was used to obtain serial LM images at 100× magnification across the full cross-section for each VR and to tile the digital images into a high-resolution montage. The contours of each VR and all myelinated nerve fibers were segmented using Fiji ImageJ or Neurolucida 360 software to determine the VR cross-sectional area as well as the cross-sectional area, perimeter, and centroid for each myelinated fiber (Bartmeyer et al., 2021). A shape-adjusted ellipse approach was used to correct for varied nerve fiber dispersion angles, calculate individual fiber diameters, and assign individual myelinated VR fibers to autonomic and motor functional categories (Bartmeyer et al., 2021).

### Identification of nerve fiber clusters

A nerve fiber cluster was considered as an area with higher than expected density of fibers compared to a random distribution for the same 2D space. For the calculation of nerve fiber clustering, all vascular spaces, i.e., holes, were first digitally subtracted from the endoneurium space in all VRs. Considering that infinity point patterns may satisfy a uniform distribution of fibers for all individual L6-S3 VRs, a total of 1,000 computational simulations were used to mimic homogenously distributed nerve fibers in each VR. Each simulation uses a uniform distribution to randomly generate *n* point positions within the region of interest, and each point represents a single nerve fiber within the VR. Interpoint distances (*d*) are used to compare the clustering degree of the points in the simulated patterns against the interpoint distances in the ground truth data for each VR. For the simulated values, for each simulation (s) the average interpoint distance (*d^s^*) is calculated as $d^{s}=\sum_{i}d_{\mathrm{ik}}/n$ , where *d*_ik_ is the distance of each point *i* to the *k*th-nearest neighbor (the present study was performed with *k *= 50). The set of all average interpoint values is used to compose a histogram plot, and the average distance for the uniform distribution (*U*) is expressed by $D_{U}=\sum_{s}d^{s}/s$ . The *k*th-nearest neighbor distance was also determined for all nerve fibers in each VR as ground truth data. For each VR segmental level, the average distance for each category, $D_{G}=\sum_{i}d_{\mathrm{ik}}/n_{G}$ , where *D_G_* is the average distance for a group of VRs based on segmental level, and *n_G_* is the number of individuals in each VR group.

### Comparisons of simulation and dataset distances

The cross-sectional area and shape of VRs as well as the number of nerve fibers within each VR will influence interpoint distances within each nerve root. These variables will equally affect the simulated and ground truth interpoint distances for an individual VR, but the interpoint distances may vary between different VRs. To address this potential limitation for comparisons between samples, the use of standard deviations (SDs) is proposed as a measure of similarity between the interpoint distances for the simulation-derived uniform distribution (*U*) and the ground truth dataset (*G*) for each VR. The new measure is calculated as follows:

$$SD_{\mathrm{UG}}=\frac{D_{G}-D_{U}}{\sigma_{U}}$$

The value of SD_UG_ represents the calculated similarity between the interpoint distances for the uniform distribution (*U*) and ground truth distribution (*G*) of an individual VR, and *σ_U_* is the standard deviation of the interpoint distances in the simulations. The likelihood that the ground truth interdistances represent a random distribution is expressed in the value of SD_UG_ for an individual VR. The SD_UG_ value is calculated separately for each fascicle for VRs with multiple fascicles and is represented by the weighted average for the fascicles and calculated as:

$$SD_{\mathrm{UG}}=\frac{\sum_{k}(n_{k}{\mathrm{SD}}_{\mathrm{UG}}^{k})}{\sum_{k}n_{k}}\;\;$$

In the above formula, *n_k_* represents the number of fibers in fascicle *k* and SD*^k^*_UG_ is the similarity index of the fascicle *k*. Standard deviation values may also be calculated for nerve roots, which are composed of more than one fascicle. Negative values of SD_UG_ represent nerve fibers groups, which are physically closer than the points in the simulations, meaning they are clustered. Positive values of SD_UG_ represent cases where the distribution of the nerve fibers is sparser than the expected value, meaning that the fibers are disperse when comparing with the points in the simulation. Separate values for SD_UG_ were calculated for the PPN fiber, αMN fiber, and γMN fiber subpopulations of the individual primate L6-S3 VRs in the present study.

### Cluster visualization and delineation

To visualize and delineate clusters quantitatively, the algorithm OPTICS, Ordering Points to Identify Cluster Structure, (Ankerst et al., 1999) was used to define nerve fiber criteria for cluster inclusion. The use of OPTICS allowed identification of clusters with varied shapes, and the algorithm was able to identify clustered and disregard none-clustered nerve fibers during the analysis process. The OPTICS algorithm creates a hierarchical structure defining all the meaningful clusters in the dataset based on the distance between individual nerve fibers as the interpoint distance. The decision of the degrees of clustering is given in a post-processing phase where the user sets the threshold interpoint distance, called reachability distance. Once the reachability distance is defined, the OPTICS returns the number of clusters and identifies the individual nerve fibers, which contribute to each cluster. The OPTICS algorithm was applied to all fibers within each VR and to subgroups of PPN fibers, αMN fibers, and γMN fibers. The reachability distance was selected based on the interpoint distances generated by the simulations for each VR. The user also defined the minimal number of nerve fibers needed to meet the reachability distance in order to represent a cluster. A minimum a four nerve fibers to form a cluster and an interpoint distance to reflect a threshold of −4 SD_UG_ were applied in the present study as criteria for a nerve fiber cluster.

### Computational aspects

The proposed analysis flow was coded in R (v3.6.3) using the Rstudio (v2021.09.0+351) interface. It used the libraries “spatstat” (v2.2-0) and “dbscan” (v1.1-8). The script is available on https://github.com/petrabartmeyer/2D_distribution.

### Code accessibility

The code described in the paper is freely available online at https://github.com/petrabartmeyer/2D_distribution. The code is available as Extended Data.

10.1523/ENEURO.0009-23.2024.d1 Extended Data 1. Code for 2-D distribution analysis. Download Extended Data 1, DOCX file.

### Statistics

The statistical analysis was performed using R (v3.6.3). All data sets are presented as mean ± standard error (SE). The nonparametric Mann–Whitney, Determination Coefficient, and Correlation Coefficient tests were applied for calculating statistical differences between groups (Table 1).

**Table 1.** Statistical table of all analyses

	Structure of the data	Type of test	Power, confidence interval
Fig. 1*D,E*	nonparametric distribution	Mann–Whitney	95%
Fig. 2*D*	nonparametric distribution	Mann–Whitney	95%
Fig. 3*C*	nonparametric distribution	Mann–Whitney	95%
Fig. 4*A,B*	nonparametric distribution	Mann–Whitney	95%
Fig. 9*A,B*	nonparametric distribution	Determination coefficient	95%
Fig. 9*C*	nonparametric distribution	Correlation coefficient	95%

## Results

**Figure 1.**
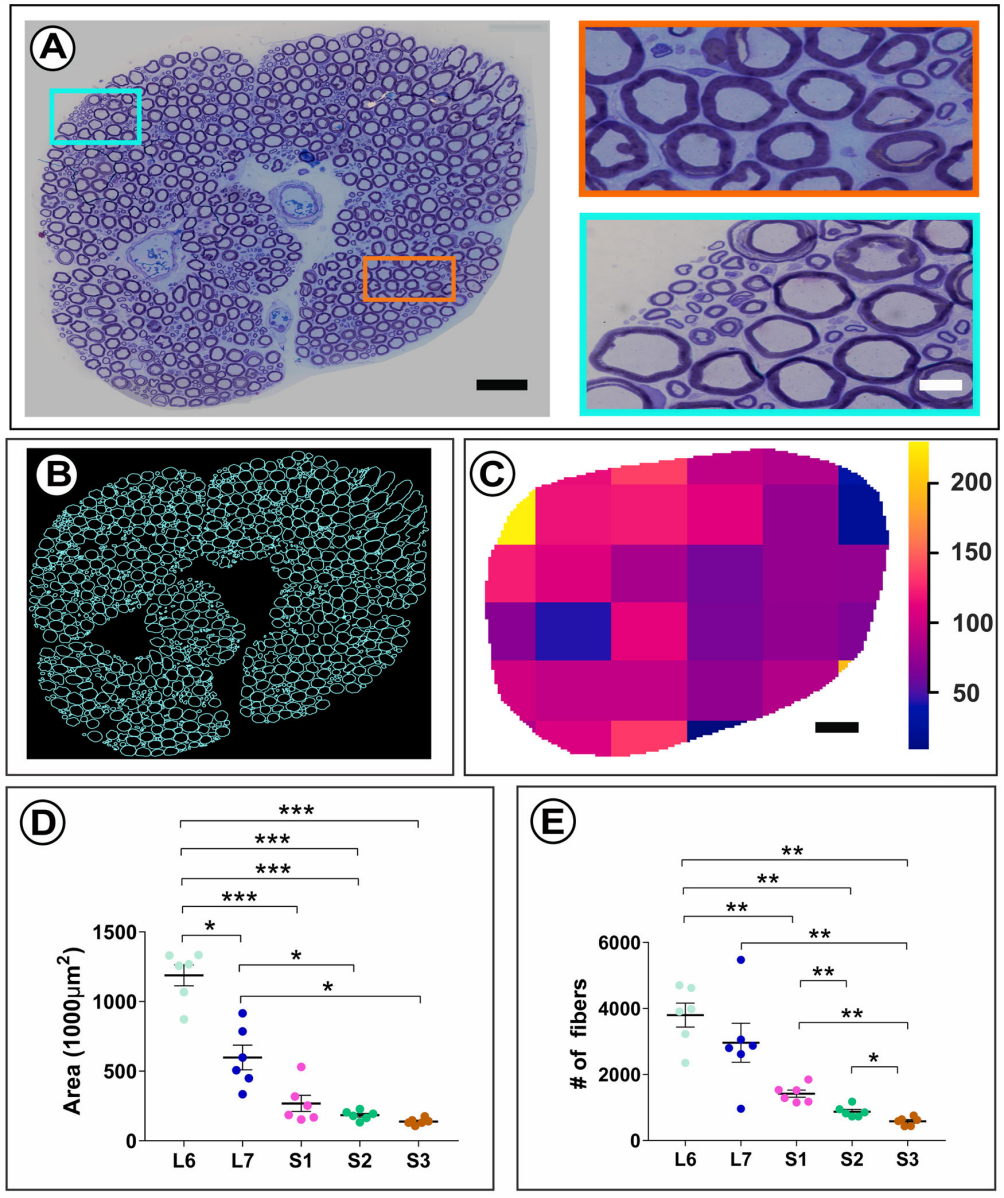
Segmentation and distribution of myelinated axons. ***A***, Light micrograph of a plastic resin-embedded and toluidine-blue stained transverse section of an S1 ventral root (VR) in a rhesus macaque shows cross-sectional distribution of myelinated fibers. Boxed areas show size heterogeneity of myelinated axons in close-up views. ***B***, A total of 1,152 myelinated axons were manually segmented to allow for digital display and quantitative analysis of all nerve fibers. ***C***, Intensity map for the localization of myelinated axons over a grid of 6 × 6 squares. The heat map display reflects the number of nerve fibers per square. Note extensive variability in nerve fiber density across the VR cross-sectional surface. ***D,E***, Displays of L6-S3 VR size indicators show a significant decrease for the cross-sectional area and number of myelinated fibers in the caudal direction. Statistics were performed as paired analysis between segmental levels with *, **, and *** indicating *p* < 0.05, *p* < 0.01, and *p* < 0.001, respectively. Scale bar is 50 µm for overview in ***A***, 10 µm for boxed area in ***A***, and 50 µm in ***C***.

For identification and mapping of autonomic fibers in lumbosacral ventral roots (VRs), L6-S3 VRs were procured in female rhesus macaques (*n* = 6) and embedded in plastic resin. Toluidine blue-stained transverse sections of the VRs allowed for high-resolution LM imaging of myelinated nerve fibers (Fig. 1*A*). The marked individual variation of myelinated fiber size was noted across the VR cross-sectional areas (Fig. 1*A*) and corresponds to established populations of preganglionic parasympathetic, somatic γ-motor, and somatic α-motor fibers in rhesus macaques (Bartmeyer et al., 2021). To allow for digital localization and detailed morphological analysis of both autonomic and motor fibers in individual VRs, manual segmentation of all myelinated fibers was performed for the L6-S3 VRs in all animals (Fig. 1*B*). Individual VRs were next projected onto a grid of 6 × 6 squares. The number of centroids, corresponding to segmented myelinated fibers, was determined for each square. Intensity maps of centroid localizations across the grid of squares suggested uneven distribution of myelinated nerve fibers across the 2D surface area of entire individual VRs (Fig. 1*C*). Additional morphological characterization of the L6-S3 VRs showed a progressive and significant decrease in size with regard to both VR cross-sectional area and total number of myelinated axons in a caudal direction (Fig. 1*D,E*).

**Figure 2.**
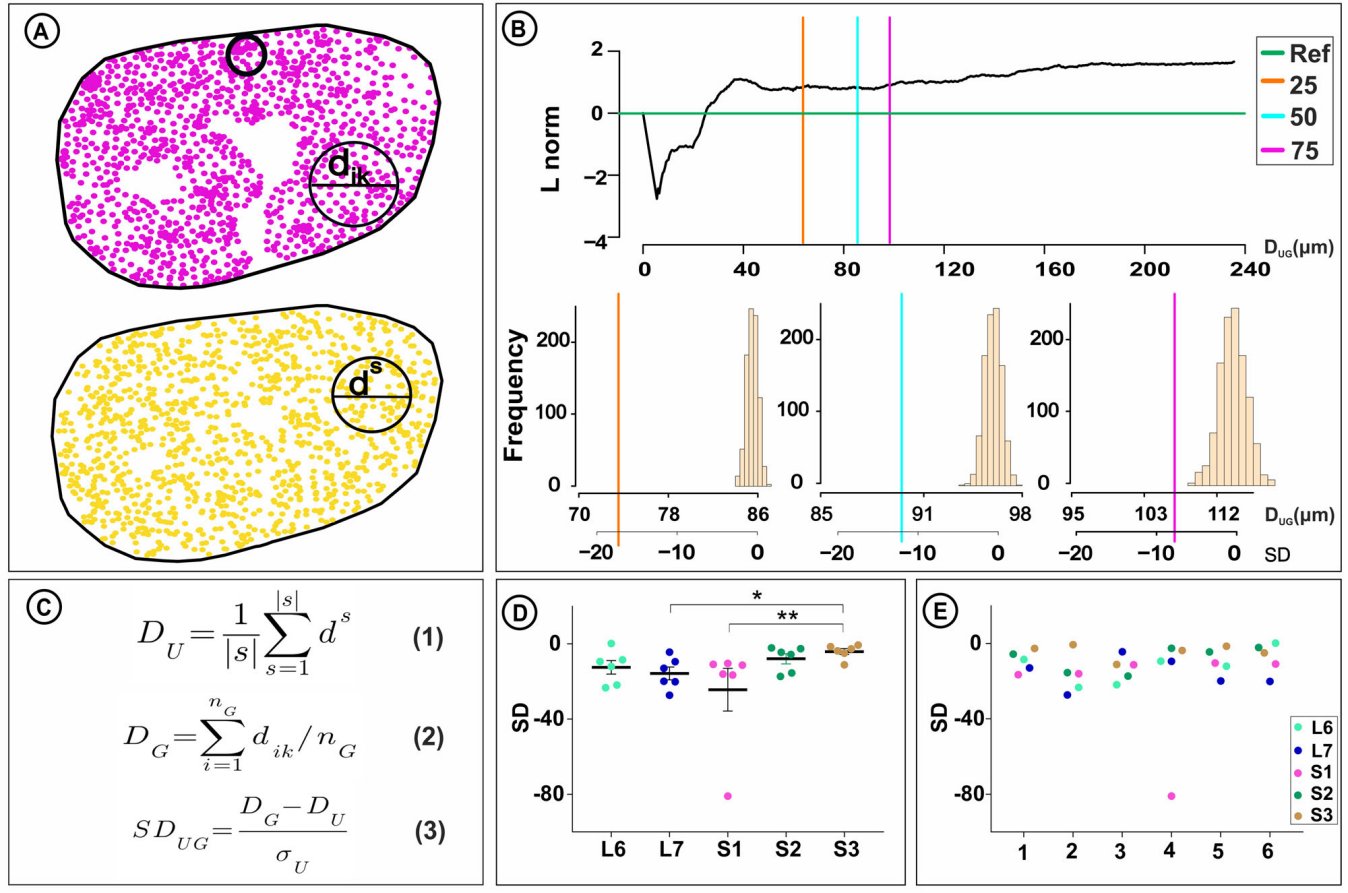
Generation of a 2D distribution map for determination of nerve fiber clustering in an S1 VR in rhesus macaque. ***A***, The 2D distribution of nerve fiber centroids for all myelinated axons (*n* = 1,152) of an S1 VR in rhesus macaque is shown in magenta as ground truth data. An unlabeled circle provides an example of an area with apparent increased nerve fiber density, i.e., clustering. A representative example of a random 2D distribution for the same myelinated axons is shown in golden color display. To determine the likelihood for the observed nerve fiber distribution to represent a chance event, a quantitative assessment of clustering was performed. Hence, a total of 1,000 consecutive random distributions of all nerve fiber centroids was performed to calculate the average distance between each nerve fiber to its 50th closest neighboring fiber for each experiment (DG). The distance corresponds to the radius of a circle, which includes the most proximal 50 neighboring fibers. ***B***, The L-function was used as an established strategy to assess point pattern structures by comparing the number of structures inside a circle of known diameter (Baddeley et al., 2000). Here, the normalized L-function (L norm) was used to study the general distributions of myelinated fiber centroids of each VR sample and to guide the selection of the optimal *k*th-nearest neighbor used for the analysis. The *x*-axis shows the diameter of a circle, and the *y*-axis shows the normalized L-function. The green horizontal line represents the expected behavior for a random point pattern following a Poisson distribution. The black line shows the normalized L function for the sample S1 VR. L-function values above the blue line indicate presence of circle spaces encompassing more fibers than expected in a random Poisson distribution. Here, nerve fiber clustering is detectable for circle spaces with a diameter exceeding approximately 20 µm, influenced by the myelinated axon size range with the largest fibers encountered in this VR being approximately 20 µm in diameter. Orange, blue, and magenta vertical lines intercept the normalized L-function and *x*-axis, indicating the mean circle diameter when calculating the distance to the nearest 25th, 50th, or 75th neighbor, respectively. Use of the 50th neighbor for determining possible clustering nerve fiber behavior, as it represented a secure number across all 30 VRs in the present sample. ***C***, Summary of formulas for determination of SD as an expression of centroid distribution to have occurred by chance. See Materials and Methods section for formula details. ***D***, Comparison of SD as an indicator of nerve fiber clustering between and within animals. Note extensive variability between VR segments within each animal. There was no significant difference for SD between the individual VR segments. Display of SD as an indicator for nerve fiber clustering in L6-S3 VRs within each rhesus macaque (*n* = 6). Note extensive variability between segmental levels for each animal. There was no significant difference for SD between animals.

To establish a quantitative 2D map of segmented nerve fibers and identify nerve fiber clustering within the full cross-sectional area of individual VRs, a new analytical tool was developed. Here, an ideal and stable equilibrium of distances was first determined for nerve fibers within a sample VR. For this purpose, the SPATSTAT R Package was used to perform 1,000 consecutive random distributions of the nerve fiber centroids within the VR, and the average distance between each nerve fiber and its 50th closest neighboring fiber was calculated for each experiment (DU). This distance represented the radius of a circle, which encompassed the 50 most proximal neighboring fibers. A normal distribution histogram for the distances was formed, based on the average distance to the 50th neighbor from each random distribution experiment (*n* = 1,000) (Fig. 2*A,B*). Next, the SPATSTAT R Package was used to determine the distance between each nerve fiber and its 50th closest neighboring fiber for the ground truth distribution (DG) of fibers in the sample VR, thereby allowing for a quantitative comparison of calculated distances with the normal distribution histogram (Fig. 2*A–C*).

The above choice of a suitable number of closest nerve fiber neighbors for each fiber was determined with the use of L-function as an established strategy to assess point patterns within a circle of known diameter (Baddeley et al., 2000). The normalized L-function was used to study the general distributions of myelinated fiber centroids of each VR sample and to guide the selection of the optimal for the *k*th-nearest neighbor used for the analysis (Fig. 2*B*). Nerve fiber clustering was detectable for circle spaces with a diameter larger than approximately 20 µm, influenced by the myelinated axon size range with the largest fibers encountered in the L6-S3 VRs being approximately 20 µm in diameter (Fig. 2*B*). Intercepts of the normalized L-function was determined for each sample VR when calculating the distance for each nerve fiber to the nearest 25th, 50th, and 75th neighboring fiber (Fig. 2*B*). Use of the 50th neighbor for determining possible clustering nerve fiber behavior was selected, as it represented a secure number across all 30 VRs in the present sample.

The primary outcome measure for the proposed tool is distance to the *k*th neighboring fiber. This metric expression may be affected by VR size and overall fiber compositions. To determine the likelihood for the ground truth 2D distribution of nerve fibers to reflect a chance occurrence and allow for comparisons between VR samples, the observed mean nerve fiber distance to the 50th closest neighboring nerve fiber was expressed as the number of standard deviations (SDs) from the corresponding mean distance value of the normal distribution histogram (Fig. 2*B,C*). An extensive variability in the 2D distribution of nerve fibers was seen within and between the L6-S3 VR levels (Fig. 2*D*). The mean SD for the L6 VRs was −12.5 ± 3.6 (*n* = 6), and the mean SD for the L7 VRs was −15.7 ± 3.4 (*n* = 6) and significantly lower than the mean SD of −4.1 ± 1.5 for the S3 VRs (*n* = 6) (*p* < 0.05) (Fig. 2*D*). The mean SD for the S1 VRs was −24.4 ± 11.4 (*n* = 6) and significantly lower than the corresponding mean SD for S3 VRs (*n* = 6) (*p* < 0.05), whereas the mean SD for the S2 VR2 was of −7.9 ± 2.7 (*n* = 6) (Fig. 2*D*). There was also extensive variability in the degree of nerve fiber clustering between the L6-S3 VR segmental levels for each animal but no significant difference in the 2D distribution for nerve fibers between animals (Fig. 2*E*).

**Figure 3.**
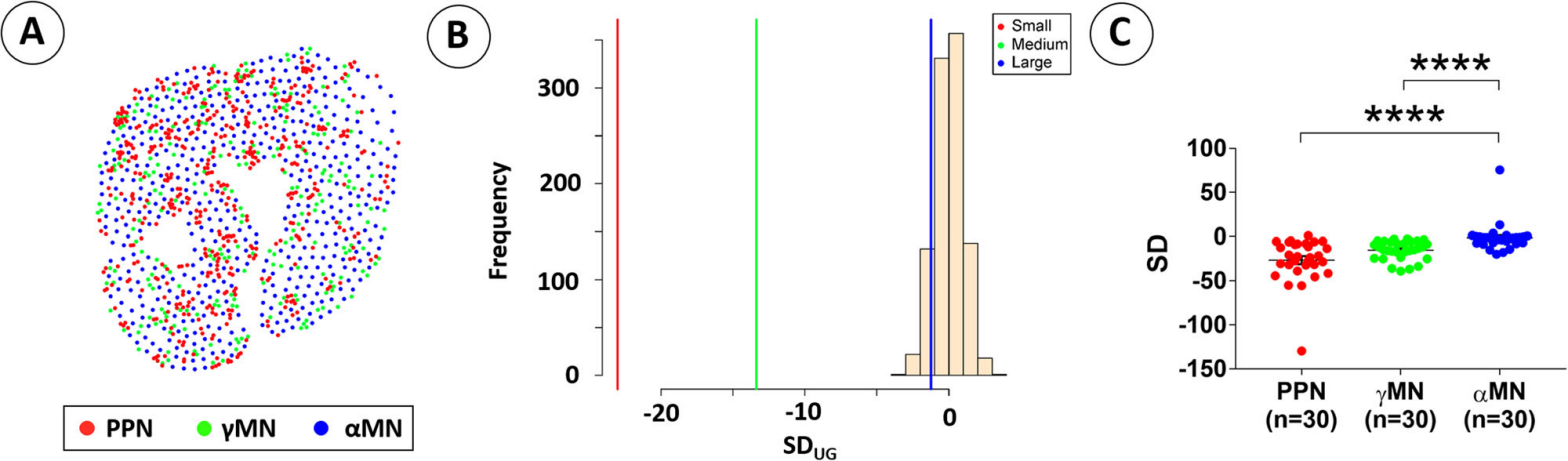
Generation of a 2D map of nerve fiber clustering for subpopulations of fibers in an S1 VR. ***A***, Myelinated fibers were divided into subpopulations with fiber diameters of 0–4 µm, >4–10 µm, and >10 µm representing PPN fibers, γ-motor fibers, and α-motor fibers, respectively (Bartmeyer et al., 2021). The distance between the centroid for each nerve fiber to the 50th closest neighbor was also determined as ground truth distribution for each of the three subpopulations of nerve fibers. The SD was −26.6 ± 4.6 for PPN fibers, −15.4 ± 1.9 for γMN fibers, and −1.3 ± 2.9 for αMN fibers in the sample S1 VR. ***B***, The SD for PPN fibers (*n* = 30) was not different from corresponding SD for γMN fibers (*n* = 30) (*p* = 0.0514) but significantly lower than the SD for αMN fibers (*n* = 30) (*p* < 0.001). The SD for the γMN fibers was also significantly lower than the SD for αMN fibers (*p* < 0.01).

To determine whether the degree of myelinated fiber clustering in individual L6-S3 VRs may be affected by the individual VR composition of different fiber types, subpopulations of fibers were studied separately in each L6-S3 VR from six rhesus macaques. In each VR (*n* = 30), myelinated fibers were divided by fiber diameter into separate groups as small (0–4 µm), medium (>4–10 µm), and large (>10 µm) fibers, which correspond to preganglionic parasympathetic neuron fibers (PPN fibers), γ-motor neuron fibers (γMN fibers), and α-motor neuron fibers (αMN fibers), respectively (Fig. 3*A*). The mean SD for PPN fibers was −26.6 ± 4.6 (*n* = 30) and not significantly lower than the corresponding mean SD for γMN fibers of −15.4 ± 1.9 (*n* = 30) (*p* = 0.0514) but significantly lower than the corresponding SD for αMN fibers −1.3 ± 2.9 (*n* = 30) (*p* < 0.0001) (Fig. 3*B,C*). The mean SD for γMN fibers was also significantly lower than the corresponding SD for αMN fibers (*p* < 0.0001) (Fig. 3*B,C*).

**Figure 4.**
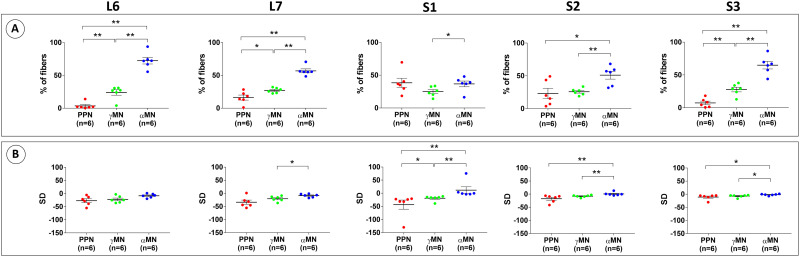
Proportions and clustering of nerve fiber subpopulations in lumbosacral VRs. ***A***, Relative proportions of PPN fibers, γMN fibers, and αMN fibers vary across L6-S3 VRs in rhesus macaques with the S1 VR being the dominant segmental level for PPN fibers. The proportion of PPN fibers was indistinguishable from the corresponding proportions of γMN fibers and αMN fibers in the S1 VR, whereas the percentage of αMN fibers was significantly higher than the corresponding percentage of PPN fibers in the adjacent L6, L7, S2, and S3 VRs. ***B***, SD calculations for individual VRs and fiber subpopulations showed marked variations both within and between fiber types. However, the S1 VRs showed the smallest standard error but also exhibited over 10% of the fiber population in the PPN fiber category.

With regard to autonomic fiber presence within the L6-S3 VRs, the S1 VR was the dominant segmental level with PPN fibers representing 38.4 ± 7.0% of all myelinated fibers (*n* = 6) (Fig. 4*A*). The corresponding proportions of PPN fibers in adjacent VRs were 3.6 ± 2.1% for L6 VRs (*n* = 6), 16.1 ± 3.8% in L7 VRs (*n* = 6), 23.2 ± 7.8% in S2 VRs (*n* = 6), and 7.4 ± 2.8% in S3 VRs (*n* = 6) (Fig. 4*A*). The S1 VRs was the only segmental level with over 10% PPN fiber representation across all animals (*n* = 6) with a markedly sparse population of PPN fibers in immediately adjacent VRs in some primates (Fig. 4*A*). There was no statistical difference between the percentages of PPN fibers compared to αMN fibers in S1 VRs (Fig. 4*A*). In contrast, the percentage of PPN fibers was significantly lower than the percentage of αMN fibers in the L6, L7, and S3 VRs (*p* < 0.01) and in the S2 VRs (*p* < 0.05) (Fig. 4*A*). The degree of clustering for the subpopulation of nerve fibers within the PPN fiber size range also varied extensively between nerve roots. The mean SD was −27.2 ± 7.3 for L6 VRs (*n* = 6), −33.8 ± 8.1 for L7 VRs (*n* = 6), −43.4 ± 17.4 for S1 VRs (*n* = 6), −17.4 ± 5.8 for S2 VRs (*n* = 6), and −11.0 ± 4.0 for S3 VRs (*n* = 6) (Fig. 4*B*).

**Figure 5.**
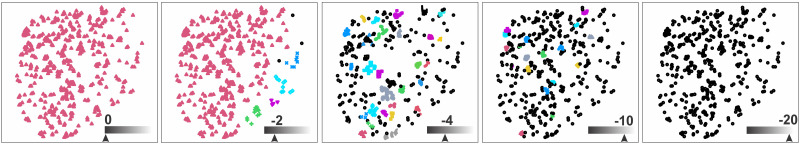
Influence of distance criteria on the identification of nerve fiber clusters in VRs. A minimum of four fibers was selected to represent a cluster of nerve fibers. The number and size of nerve fiber clusters also depend on the selected threshold number of SDs for demonstrating clusters. SDs of 0, −2, −4, −10, and −20 were selected and based on a total of 1,000 consecutive random distributions of all nerve fiber centroids to calculate the average distance between each nerve fiber to its 50th closest neighboring fiber for each experiment (Fig. 2). For an SD of 0, a single nerve fiber cluster is identified and includes all nerve fibers. A decreasing likelihood of a chance distribution was reflected in the ground truth sample when SD was selected at −2, −4, and −10, as the number of clusters gradually increased to 7, 21, and 29, respectively, with a concurrent decrease in the cluster size. However, no clusters were identified when the threshold is at −20 SDs. Nerve fiber clusters within any VR are indicated by different colors, and fibers not included in a cluster are shown in black.

In order to determine presence or the degree of nerve fiber clustering in a VR or peripheral nerve, the definition of what represents a cluster needs to be considered. Here, we present a new analytical tool that allows for a customized and nonbinary cluster definition based on two variables. First, the minimum number of fibers needed to form a cluster is chosen. Second, the SD threshold is selected as the number of negative SDs and is based on an expression of the maximum distance allowed between individual pairs of fibers for their inclusion as neighbors in a cluster. It is noted that the SD is determined individually in each VR after performing a total of 1,000 consecutive random distributions of all nerve fiber centroids within the same VR and calculating the average distance between each individual nerve fiber and its 50th closest neighboring fiber to form a normal distribution histogram (Fig. 2*A,B*). Both variables are investigator-determined, thereby allowing a range from loose to strict criteria for the nerve fiber cluster definition in customized analysis (Fig. 5). The calculation of SD for any ground truth distribution of nerve fibers in a VR or peripheral nerve determines the likelihood for a given distribution to represent a chance occurrence.

**Figure 6.**
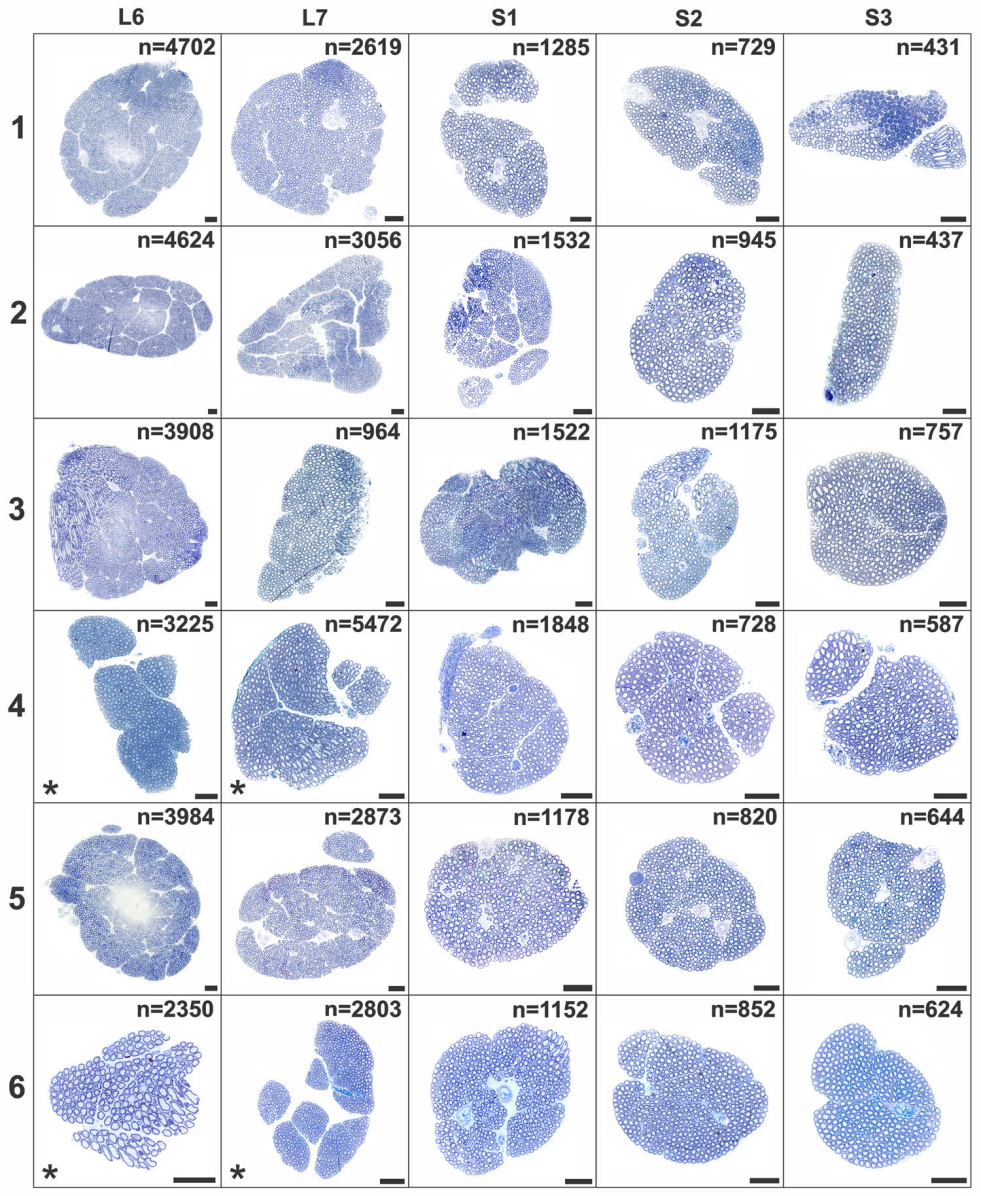
Light micrographs of plastic embedded and toluidine blue-stained L6-S3 VR cross-sections in rhesus macaques (*n* = 6). Note heterogeneity in VR shape, size, and fascicular organization. The total number of myelinated axons is shown for each VR, and * indicates only representative fascicles shown for select VRs, as fascicles were dispersed spatially in the plastic resin during the embedding process. Scale bar = 100 µm.

**Figure 7.**
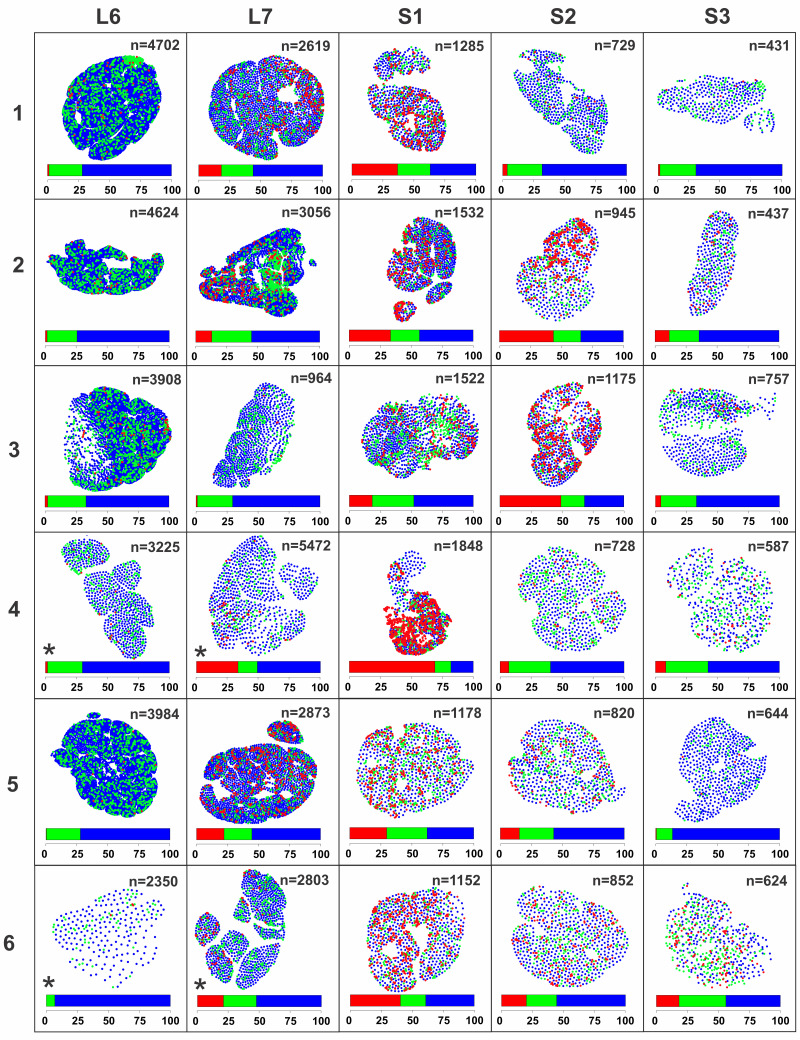
Display of digitally segmented myelinated axons in L6-S3 cross-sections of VRs in rhesus macaques (*n* = 6). The presentation corresponds to the same VRs and orientation as the toluidine blue-stained sections in Figure 6. Three populations of myelinated fibers are identified in diameter ranges of 0–4 µm, >4–10 µm, and >10 µm, representing PPN (red), γ-motor (green), and α-motor (blue) fibers, respectively, and all measurements were size corrected for varied axonal dispersion angles using the shape-adjusted ellipse approach (Bartmeyer et al., 2021). A color-coded bar indicating proportions of the three fiber types is included for each VR. For the *VR the color-coded bar presents the proportion of fibers in the entire VR, not only in the displayed fascicle. Note varied relative composition of the three fiber types between segmental levels in single animals and within a single segmental level between animals. Also note apparent clustering of small fiber populations in VRs with larger populations of PPN fibers. The total number of myelinated axons is shown for each VR, and * indicates only representative fascicles shown for select VRs as fascicles dispersed spatially in plastic resin during embedding process.

**Figure 8.**
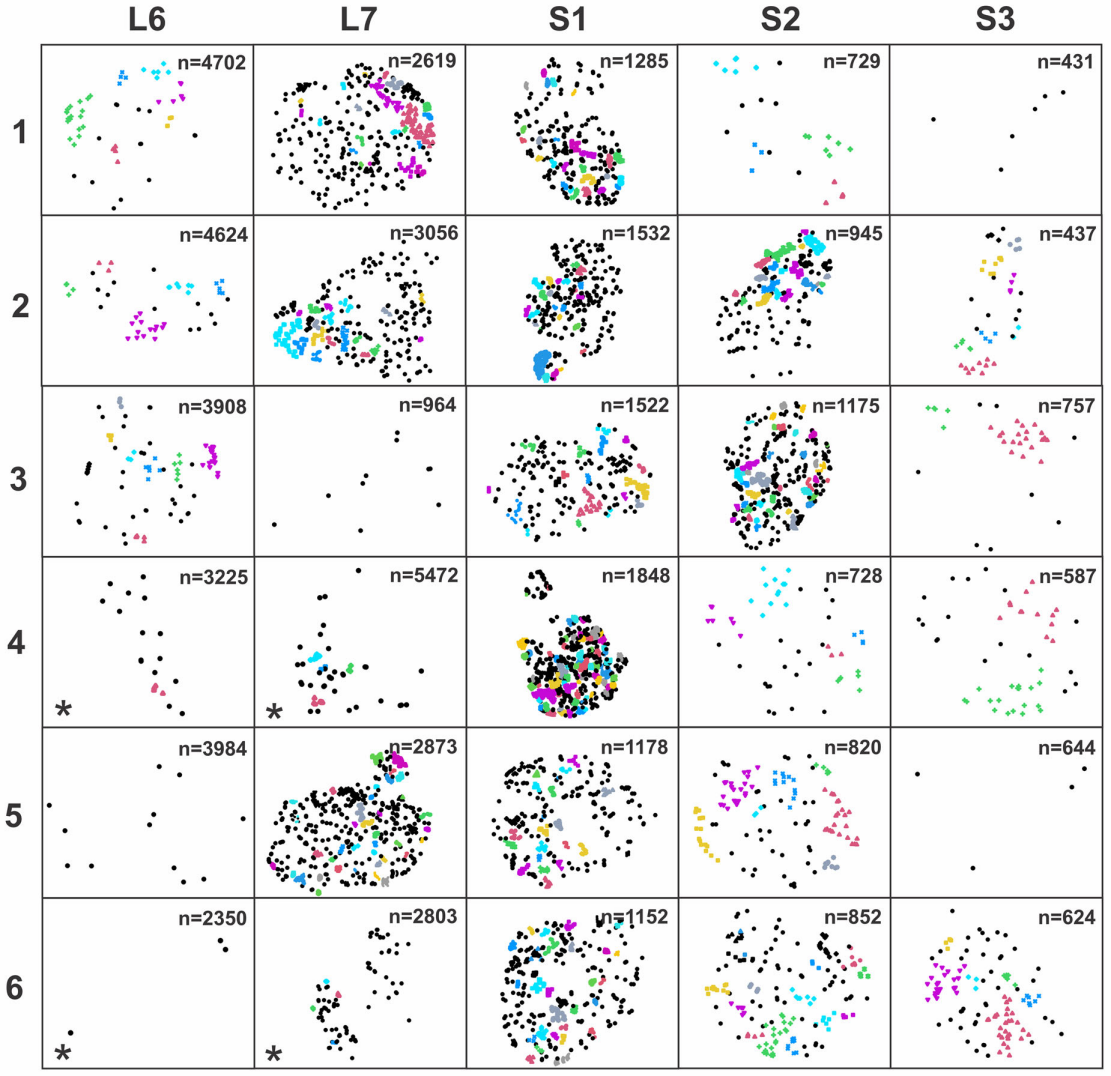
Mapping of PPN nerve fiber clusters in cross-sections of L6-S3 VRs in rhesus macaques. The display corresponds to the same VRs and orientation as presented in Figures 6 and 7. Varied degrees of cluster formation within and between animals is shown with a minimum of four myelinated fibers being required to form a cluster and the threshold criteria set at −4 SDs to indicate outcome as significant and unlikely the result of a chance distribution. For all VRs, groups of PPN nerve fibers included in separate clusters are shown in different colors, and fibers not participating in a cluster are shown in black.

Our proposed new tool was next applied for 2D distribution analysis of PPN nerve fiber clusters. Quantitative analysis of toluidine blue-stained light micrographs of the L6-S3 VRs (*n* = 6) showed varied total numbers of myelinated fibers both within and between the VR segmental levels (Fig. 6). Digital segmentation of all myelinated fibers in each VR showed much varied proportions of nerve fibers corresponding to size the size ranges for PPN fibers, γMN fibers, and αMN fibers. Furthermore, there was an apparent uneven distribution of nerve fibers in the PPN size range (Fig. 7). Next, criteria for PPN nerve fiber cluster analysis were set and consisted of a minimum of four PPN nerve fibers to form a cluster and a threshold distance to the 50th closest neighboring nerve fiber corresponding to −4 SDs from the mean value of 1,000 random PPN fiber distributions. Multiple nerve fiber clusters were identified in all S1 VRs and in the majority of the immediately adjacent L7 and S2 VRs with cluster presence most prominent in VRs with a higher percentage of small myelinated fibers in PPN fiber range (Fig. 8).

**Figure 9.**
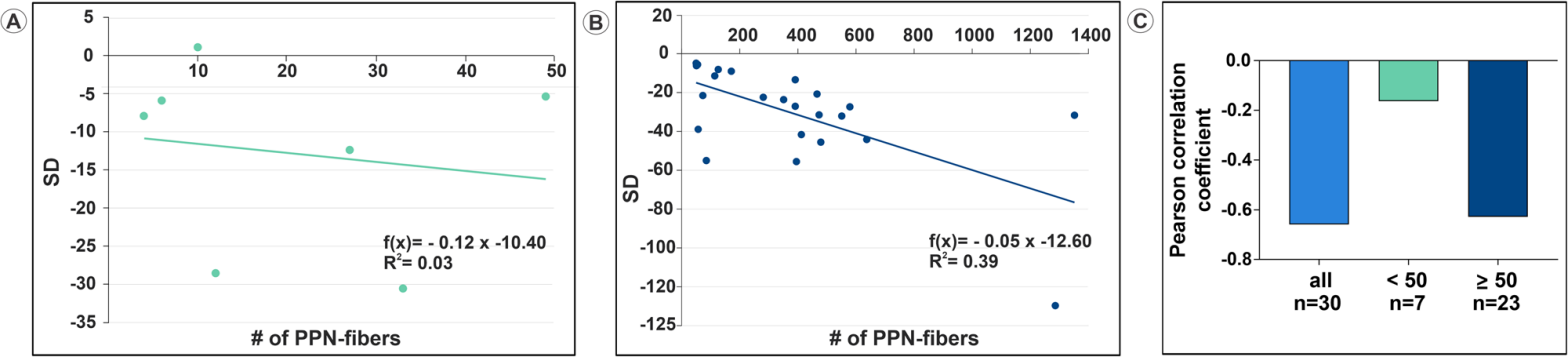
Influence of PPN fibers on clustering behavior in L6-S3 VRs (*n* = 30) of six rhesus macaques. There was a negative linear correlation between the number of PPN fibers and SD for both the subpopulation of VRs with <50 PPN fibers (*n* = 7) (***A***) and for the subpopulation of VRs with >50 PPN fibers (*n* = 23) (***B***). The effect on the dependent variable was weak for the subpopulation with <50 PPN fibers in each VR, whereas the effect on the dependent variable was moderate for the subpopulation with >50 PPN fibers per VR, as indicated by the *R*^2^ of 0.03 and 0.39, respectively. Next, the Pearson correlation coefficient was determined for both subpopulations and for all VRs combined. ***C***, A Pearson correlation coefficient of −0.16 indicated a weak negative correlation for the subset of VRs with <50 PPN fibers, whereas Pearson correlation coefficients of −0.63 and −0.66 indicated markedly stronger negative correlations for the subset of VRs with >50 PPN fibers and for the full population of all VRs combined, respectively.

We next examined the influence of PPN fibers on clustering behavior in all L6-S3 VRs (*n* = 30) for all included rhesus macaques (*n* = 6). There was a negative linear correlation between the number of PPN fibers and SD for both the subpopulation of VRs with <50 PPN fibers (*n* = 7) (Fig. 9*A*) and for the subpopulation of VRs with ≥50 PPN fibers (*n* = 23) (Fig. 9*B*). The effect on the dependent variable was weak for the subpopulation with <50 PPN fibers in each VR, whereas the effect on the dependent variable was moderate for the subpopulation with ≥50 PPN fibers per VR, as indicated by the coefficient of determination, *R*^2^, of 0.03 and 0.39, respectively. Next, the Pearson correlation coefficient was determined for both subpopulations and for all VRs combined. A Pearson correlation coefficient of −0.16 indicated a weak negative correlation for the subset of VRs with <50 PPN fibers, whereas Pearson correlation coefficients of −0.63 and −0.66 indicated markedly stronger negative correlations for the subset of VRs with ≥50 PPN fibers and for the full population of all VRs combined, respectively (Fig. 9*C*). Collectively, the correlation studies suggest that the presence of a larger population of PPN fibers in lumbosacral VRs promotes increased clustering and heterogeneity in the 2D distribution of nerve fibers.

## Discussion

A new analytical tool was proposed and allowed for morphological mapping of the projectome of digitally segmented nerve fibers within the contours of, for instance, a nerve root or fascicle in the PNS. Our approach of using 2D distributional analysis to identify and quantify fiber clusters and subpopulation interactions builds on previous studies in other biological systems. For instance, studies of tree demography and density in forestry identified that large-diameter trees influenced forest structural heterogeneity by means of competitive interactions with other tree species (Lutz et al., 2013). The notion of modeling to compare simulated and observed data showed utility for the detection of inhomogeneous distributions of viable trees with varied stem sizes across a forest area (Genet and Pothier, 2013). However, our proposed tool includes new and expanded features, including investigator-selected and customized criteria for nerve fiber heterogeneity and nonbinary identification of clusters sharing specific morphological features. Also, the use of SD allows expansion of the measure of dissimilarity for the comparison of VRs at different segmental levels.

Lumbosacral VRs in primates were used as a model system to evaluate our proposed 2D distribution tool for nerve fiber mapping in the PNS. The L6-S3 VRs in rhesus macaques feature, in varied numbers and proportions, distinct efferent populations of PPN fibers, γMN fibers, and αMN fibers (Bartmeyer et al., 2021). Our classification of nerve fibers into functional types was based on fiber size, and the size of individual nerve fibers was corrected for varied axonal dispersion angles with the use of the shape-adjusted ellipse approach (Bartmeyer et al., 2021). No corresponding physiologic recordings of e.g., evoked compound nerve action potentials to validate the distribution of nerve conduction properties are available from the same VRs, and this represents a potential limitation of the study. However, the new 2D distribution tool allowed for the quantification of nerve fiber clusters with regard to both the entire population of all myelinated fibers as well as separate cluster quantification of the different functional subpopulations. Parasympathetic efferent projections are also present within the conus medullaris portion of the spinal cord across mammalian species. For instance, PPN fibers originate primarily within the L6-S1 segments of the rat spinal cord (Coggeshall et al., 1977, 1980; Hoang et al., 2003; Akhavan et al., 2006). In cats, PPNs typically reside in two or three consecutive sacral segments of the spinal cord with most PPN fibers extending with their peripheral projections into the S2 VR (Nadelhaft et al., 1980, 1986). In the human spinal cord, PPN fibers similarly extend into the S2-S5 VRs and are most abundant in the S3-S5 roots (Hauck et al., 2009). It remains to be determined whether clusters of efferent subpopulations of autonomic and motor fibers form also in lumbosacral VRs across species.

In the present study, the analyses addressed the 2D distribution of nerve fibers within the size range of efferent PPN, γMN, and αMN fibers. It is possible that afferent nerve fibers may also be present in lumbosacral VRs (Hildebrand et al., 1997). Early studies of ventral root afferents have suggested a presence of unmyelinated fibers in lumbosacral nerve roots of rats and cats (Coggeshall et al., 1973, 1980; Clifton et al., 1976). Electrophysiologic studies of L7 and S1 VRs in cats suggested presence of ventral root afferents with conduction velocities consistent with both unmyelinated and myelinated axons (Coggeshall and Ito, 1977). In the present light microscopic study of myelinated axons, unmyelinated C fibers were excluded, but a subpopulation of myelinated sensory fibers with an overlap in size with the efferent autonomic and motor fibers cannot be excluded.

Improved understanding of efferent parasympathetic fibers, especially in human lumbosacral VRs, may provide an opportunity for improved neuromodulation strategies to reverse neurological impairments of e.g., pelvic organs. Evoked responses and therapeutic outcomes vary in experimental models and clinical studies of lower urinary tract dysfunction for sacral anterior root stimulation (Carlucci et al., 2014), spinal nerve stimulation (Carlucci et al., 2014; Kapriniotis et al., 2022), epidural spinal cord stimulation (Zhang et al., 2014; Zander et al., 2020), and transcutaneous spinal cord stimulation (Gad et al., 2018; Havton et al., 2019). Nerve root activation is common to all of these neuromodulation strategies, and a variety of anatomical factors may contribute to the varied treatment effect. Varied rostro-caudal distribution of specialized motor and PPN columns and their efferent fiber exits into lumbosacral VRs represent established anatomical variables across large mammals (Sherrington, 1892; VanderHorst and Holstege, 1997; Gross et al., 2017; Bartmeyer et al., 2021). The present study identifies heterogeneity in the degree of clustering of efferent parasympathetic fibers within individual VRs as an additional variable with the potential to influence the nerve fiber activation to electrical stimulation forms of neuromodulation.

Other PNS neuromodulation targets also feature a combination of somatic motor and autonomic fibers. Vagus nerve stimulation (VNS) may be used as a treatment option for patients with for medication-refractory epilepsy or treatment-resistant depression (Pérez-Carbonell et al., 2020; Ryvlin et al., 2021; Austelle et al., 2022). VNS is typically applied over the cervical segment of the vagus nerve, which carries motor fibers in the αMN- and γMN fiber ranges, PPN fibers, and both myelinated and unmyelinated sensory fibers (Havton et al., 2021). The cervical segment of the vagus nerve may show multiple and distinct fascicles of varied shape and size (Pelot et al., 2020; Havton et al., 2021), but possible 2D clustering of distinct morphological classes of nerve fibers based on axonal caliber and myelination remains to be determined. However, recent advances in human vagus tissue procurement, preservation, and new tools for automated segmentation and quantitative analysis of high-resolution electron micrograph montages will allow for detailed 2D mapping of fascicular nerve fiber contents (Bartmeyer et al., 2021; Havton et al., 2021; Plebani et al., 2022).

PPN fibers extend from the sacral parasympathetic nucleus in the conus medullaris, exit the spinal via lumbosacral or sacral VRs, and enter the pelvic splanchnic nerves (Coggeshall et al., 1977; Nadelhaft et al., 1986; Hoang et al., 2003; Hauck et al., 2009). In contrast to the cervical vagus nerve, the bilateral pelvic nerves, as they project to the major pelvic ganglia, carry both autonomic efferent and visceral afferent fibers but no somatic motor fibers. It remains to be determined whether 2D distribution of fibers and possible clustering of, for instance, PPN fibers may differ along the course between the spinal cord exit zone and peripheral targets. Such differences may influence emerging stimulation and response strategies for modeling of the pelvic nerve as a neuromodulation target (Eiber et al., 2021).

Electrical stimulation-evoked activation of PPNs in lumbosacral VRs may be influenced by multiple morphological factors, including ventral root cross-sectional area, axonal diameters, and nerve fiber myelination. For instance, the L6 and L7 VRs in rhesus macaques show a larger cross-sectional area compared to S1-S3 VRs in rhesus macaques (Ohlsson et al., 2013; Bartmeyer et al., 2021), and the larger VRs will include nerve fibers with a longer distance between, for instance, the position of a cuff stimulation electrode attached to the outside surface of the nerve root and the nerve fibers positioned within the mid-portion of the VR. Provided that all other potential influencing factors being equal, an increased electrode-to-fiber distance is expected to increase the activation threshold (Follett and Mann, 1986; Pelot et al., 2017). Also, myelinated fibers with smaller diameters, including PPN fibers, show slower conduction velocity and a higher threshold of electrical excitability (McPhedran et al., 1965; Gorman and Mortimer, 1983; Zajac and Faden, 1985). Information on the location of possible clustering of nerve fiber subpopulations within, e.g., fascicles, VRs or peripheral nerves may therefore guide refinement of electrical stimulation strategies for the recruitment of PPN fibers, or other select nerve fiber populations, and minimize the risks for adverse responses, including off-target effects.

### Conclusion

In conclusion, we used L6-S3 VRs in rhesus macaques as a model system to validate a new tool for 2D distribution analysis of nerve fibers in the PNS. Marked differences in cluster formation for PPN, γMN, and αMN fiber populations was shown within individual VRs as well as between adjacent segmental levels for each primate and between animals at all studied segmental levels. An increased presence of PPN fibers in VRs was positively correlated with increased nerve fiber clustering. The new tool and present findings may be of importance for the development of refined neuromodulation strategies to reverse conditions affecting visceral organs.

## Synthesis

Reviewing Editor: Nicholas J. Priebe, University of Texas at Austin Decisions are customarily a result of the Reviewing Editor and the peer reviewers coming together and discussing their recommendations until a consensus is reached. When revisions are invited, a fact-based synthesis statement explaining their decision and outlining what is needed to prepare a revision will be listed below. The following reviewer(s) agreed to reveal their identity: Chen Ran.

The initial critiques provided by reviewers and the online discussion raised a number of concerns on the manuscript in its present form. Both reviewers and the reviewing editor thought that this new experimental tool could be useful to the neuroscience community and likely extend beyond the primate autonomic nerves that you applied it to. But we did not feel that you presented sufficient evidence to show that this tool is an advance over currently available approaches. Quoting an online discussion comment "The concept of this clustering methodology is interesting and potentially promising, but more work is needed to convince me that it is actually working ... Once that step has been made the question then remains how much it will compare to current more standard methodologies." After our online discussion, the reviewers and reviewing editor came to the conclusion that more work is needed to address these issues as well as those outlined beloe. Typically, the synthesis statement for eNeuro brings review comments and online discussion together into a single statement. However when extensive more work is needed, I think it is useful for the authors to see the full comments of the individual reviewers.

Reviewer 1 -

This study provides 2D mapping of parasympathetic fibers in ventral roots. This is an understudied but important area of research. The manuscript is generally well-written and has clinical applications. There are a few points that will help the clarity and applicability of the paper as follows.

Major points:

1) It is very confusing to use the term "standard deviation" as the primary outcome measures. Standard deviation should always be a negative number, and thus the term SD is confusing. In Fig 2C, all the "SDs" are negative. Is this because the uniform distance is always going to be more than the actual distance since naturally the fibers will cluster? This point needs clarification. The authors should strongly consider a different term to describe the data because (I think) they are not actually reporting standard deviation.

2) How is overall ventral root cross sectional area normalized (regarding the fiber clustering)? What is the size variability from L6 to S3?

3) To validate the fiber clustering, the calculated fiber clusters should be compared to the micrographs and preferentially another identifier (in addition to axon size) should be used to more definitively identify the PPNs. If not possible, this should be mentioned as a study limitation.

4) Discussion could be improved if added how fiber clustering affects neuromodulation, e.g., does PPN-fiber activation depend on the location of the PPN-clusters? Like if the electrode is placed on the outside of the nerve itself, if the PPN-clusters are more centrally placed, are they harder to activate? Thickness of PPN-fiber myelination? Would higher electrical current harm other fiber types in the process of trying to activate the smaller PPN-fibers?

Minor points:

1)Figure 7A - representation slope of a linear correlation, so the y-axis label should indicate this is a "slope" rather than a correlation coefficient "R"

When all nerves were accounted for, doesn't that weak positive correlation between PPN and SD (again please choose different variable name) mean that increased PPN is associated with decreased clustering?

How was the 50 PPN-fibers threshold decided?

Because this is a slope, the subtitle for the y-axis should probably be SD/(# of PPN-fibers) since SD is y and PPN-fibers is x. The current subtitle makes it seem like the y-axis represents the number of PPN-fibers multiplied by SD, which are all quite large numbers and would be negative since the SD is often negative. This would match with Figure 7B.

Can the graphs that determined the slopes for Figure 7A be shown? Similar to the graph in Figure B?

Please provide the justification for the use of ln(x) for Figure 7B?

Please, should not use the word "slope" to describe the "regression coefficient" because that implies a straight line. As in the slope of a tangent line as a certain point on the graph.

Would also be helpful to see the Figure 7B graph shown with ln(x) on the x-axis, so we can see how the log transformation made the data look more linear.

2) Very last sentence of Results section states "[I]ncreased presence of myelinated PPN-fibers in L6-S3 VRs of rhesus macaques is associated with increased clustering and heterogeneity in the 2D-distributon of nerve fibers." Later in the discussion, this heterogeneity is referred to as heterogeneity in degree of clustering, but here in the results, this can be interpreted as heterogeneity in the fiber types, which would likely need some pie charts with the percentages of each fiber type to be in included in Figure 4.

3)In the results section "...(PPN-fibers),...(γMN-fibers), and...(α-MN-fibers), respectively [21]" has a different citation style than the rest of the paper, which also cannot be fixed by editors since "21" does not match with the reference style given.

4) Use of "2-D" in title and "2D" in rest of the paper. Please correct for consistency.

5) Clarity needed:"[A]utonomic nerve fibers may extend in the presence of varied number of somatic motor and sensory fibers in nerve roots and peripheral nerves." The word "extend" is unclear. Do the authors mean that the presence of other fiber types/calibers affects the excitation of autonomic fibers?

6) In the discussion, the sentence "For instance, PPN-fibers originate primarily within the segments of the rat spinal cord" seems incomplete. (which segments??) Next sentence on cats describes specifically "2-3 consecutive sacral segments" and next on humans specifically denotes "S2-S5".

Reviewer 2 -

In this manuscript the authors present a method for D2 distribution analyses (i.e. grouping) of nerve fibers. The rationale for pursuing this study is justified and relevant, while rather specialized. The methodology was tested and developed using ventral roots of 6 non-human primates, which specimens were collected in the past as part of root avulsion studies. The tissues were collected and processed carefully, and the heterogeneity in nerve fiber composition of these VRs puts the methodology to the test. As for the statistical modeling and interpretation of the modeling results, several queries came up:

1) The clustering model presented is based upon inter-point distances of fibers and number of fibers: isn't this clustering biased towards smaller fibers, simply as larger numbers of smaller fibers can be found closer to each other? Is that why we are seeing the clearest clustering in the panels with higher abundance of PPNs (Figure 6). Is this also why only clusters of tiny fibers remain when the distance is adjusted further (-10) as in Figure 5? Is this the reason that the methodology doesn't find as many clusters when the VRs are mainly composed of large profiles (as in the VRs with mainly aMNs and gMNs), as thresholds were set for a different "population" of nerve fibers?

2) Figure 1: the VR section is discontinuous due to vascular space and the 6x6 squares appear to capture these "holes". A grid from which the "holes, i.e. vascular space" are subtracted, similar to exclusion of empty space outside the VR, would provide a more robust representation.

While Figure 1 still serves it's purpose within the context of the narrative, the same "holes" carry forward in the modeling part as shown in Figure 2. To what extent do these "holes" color or skew the findings of the modeling study? This is especially relevant as the vascular spaces may vary among levels and monkeys.

Additional comments:

Figure 2: What do the unlabeled circles in the brown and magenta VRs represent?

Figure 2C: As the data from all 5 VRs was obtained from the same monkeys, it would be interesting to see how the data from individual monkeys behaves (connect the dots between levels).

Figure 6: It would be nice to see a similar panel of the raw images and the distribution of the aMNs, gMNs, and PPNs, to fully assess how the modeling behaves in relation to the "true" data.

## Author Response

Synthesis Statement for Author (Required):

The initial critiques provided by reviewers and the online discussion raised a number of concerns on the manuscript in its present form. Both reviewers and the reviewing editor thought that this new experimental tool could be useful to the neuroscience community and likely extend beyond the primate autonomic nerves that you applied it to. But we did not feel that you presented sufficient evidence to show that this tool is an advance over currently available approaches. Quoting an online discussion comment "The concept of this clustering methodology is interesting and potentially promising, but more work is needed to convince me that it is actually working ... Once that step has been made the question then remains how much it will compare to current more standard methodologies." After our online discussion, the reviewers and reviewing editor came to the conclusion that more work is needed to address these issues as well as those outlined below. Typically, the synthesis statement for eNeuro brings review comments and online discussion together into a single statement. However when extensive more work is needed, I think it is useful for the authors to see the full comments of the individual reviewers.

Reviewer 1 - This study provides 2D mapping of parasympathetic fibers in ventral roots. This is an understudied but important area of research. The manuscript is generally well-written and has clinical applications. There are a few points that will help the clarity and applicability of the paper as follows.

Major points:

1) It is very confusing to use the term "standard deviation" as the primary outcome measures. Standard deviation should always be a negative number, and thus the term SD is confusing. In Fig 2C, all the "SDs" are negative. Is this because the uniform distance is always going to be more than the actual distance since naturally the fibers will cluster? This point needs clarification. The authors should strongly consider a different term to describe the data because (I think) they are not actually reporting standard deviation.

We agree that our original description of the tool was unnecessarily confusing, and we have therefore introduced distance, a metric outcome measure, as the primary outcome measure for our proposed 2D mapping tool. We expect that the use of distance, expressed in µm, will make the tool more conceptually accessible for users. The distance calculation also represents a required intermediary step for the calculation of the standard deviation (SD), a secondary outcome measure, as is also shown in the formulas presented in the original and revised Figure 2. Our updated tool presentation will first provide users with the metric measurement of distance as the primary outcome measure and the option to next determine SD as an additional outcome measure. We have updated all figures in the revised manuscript to display both distance and SD as outcome measures. We have also clarified that the SD outcome measure provides an expression of how likely it is that the observed 2D distribution of nerve fibers is the result of a chance occurrence. In addition, the SD calculations allow for standardized outcome comparisons between nerve roots (or peripheral nerves) with different cross-sectional areas and varied numbers of nerve fibers. We have included additional text to clarify these outcome features provided by the SD calculations.

The expression of SD as a negative or positive value in our sample of nerve roots corresponds to whether the observed mean distance to the 50th closest neighbor is decreased or increased, respectively, compared to the mean distance to the 50th closest neighbor for 1,000 consecutive random distributions. For instance, a calculated SD of -6.5 for a ventral root sample suggests increased clustering of nerve fibers. However, the calculated SD does not suggest that it is impossible for the outcome to be a result of chance, but the probability is low. The use of SD as a standardized outcome measure is more suitable than our primary outcome measure of distance for making clustering behavior comparisons between nerve roots or peripheral nerves within the same animal as well as between animals of the same or different species.

2) How is overall ventral root cross sectional area normalized (regarding the fiber clustering)? What is the size variability from L6 to S3? We have added to the Results quantitative information on size variability for the L6-S3 ventral roots in the form of cross-sectional area and the number of myelinated fibers for ventral roots. We recognize that varied ventral root cross-sectional area and the number of nerve fibers for each root may influence the distance to the 50th closest nerve fiber. To normalize for these possible size-related influences on clustering and to allow for comparisons between different segmental levels and animals, we calculate, in addition to the mean distance to the 50th nearest neighboring nerve fiber for all individual nerve fibers, also the corresponding mean SD for comparison with the distribution of 1,000 consecutive random distributions for all nerve fibers within each root. We have added additional clarification on this tool feature provided by the SD component in the Discussion.

3) To validate the fiber clustering, the calculated fiber clusters should be compared to the micrographs and preferentially another identifier (in addition to axon size) should be used to more definitively identify the PPNs. If not possible, this should be mentioned as a study limitation.

We agree and have expanded the Results section with two additional Figures, which both include displays of the L6-S3 ventral roots from all six animals to validate the results and to provide full transparency of the presentation. The added (new) Figure 6 displays the light micrographs of plastic resin-embedded semi-thin sections of all L6-S3 VRs after toluidine blue staining. The added (new) Figure 7 shows the 2-D distribution of α-MN, γ-MN, and PPN fibers after fiber segmentation. The added (new) Figure 8 shows mapping of PPN fibers in clusters.

For the present study, we use fiber size as the morphological identifier for the different functional types of nerve fibers in the L6-S3 ventral roots. In an earlier study on nerve fiber size corrections in the setting of varied nerve fiber disperse angles, also based on the same archival nerve root material used in the present study, we have identified distinct fiber size populations that correspond to the α-MN, γ-MN, and PPN fibers using a statistical approach (Figure 3, Bartmeyer et al., Scientific Reports, 2021). Unfortunately, we do not have any nerve conduction recordings from these nerve roots to compare, for instance, compound nerve action potentials (CNAPs) with our present morphological findings. Surprisingly, we have not identified any studies in the literature with direct comparisons between nerve conduction properties and detailed morphological size determinations of individual nerve fibers in nerve roots or peripheral nerves from the same subjects or animals. Anatomical validation based on transmitter contents is also with limitations, as PPNs as well as α- and γ-motor fibers are all cholinergic. We have expanded the Discussion on this broader topic and also mentioned the lack of direct correlative information between peripheral conduction properties of PPNs and comprehensive morphological studies of individual fibers as a study limitation.

4) Discussion could be improved if added how fiber clustering affects neuromodulation, e.g., does PPN-fiber activation depend on the location of the PPN-clusters? Like if the electrode is placed on the outside of the nerve itself, if the PPN-clusters are more centrally placed, are they harder to activate? Thickness of PPN-fiber myelination? Would higher electrical current harm other fiber types in the process of trying to activate the smaller PPN-fibers? We thank the reviewer for this question. We agree, and we have added a paragraph in the Discussion to review how morphological features, including fascicle, nerve root, or peripheral size, fiber size and myelination, clustering, and distance to stimulating electrodes, as well as electrode features and electrical stimulation strategies, may affect fiber recruitment during neuromodulation.

Minor points:

Reviewer 1 provided several suggestions for improving the display and presentation of Figure 7:

Figure 7A - representation slope of a linear correlation, so the y-axis label should indicate this is a "slope" rather than a correlation coefficient "R"; 2) When all nerves were accounted for, doesn't that weak positive correlation between PPN and SD (again please choose different variable name) mean that increased PPN is associated with decreased clustering?; 3) Because this is a slope, the subtitle for the y-axis should probably be SD/(# of PPN-fibers) since SD is y and PPN-fibers is x. The current subtitle makes it seem like the y-axis represents the number of PPN-fibers multiplied by SD, which are all quite large numbers and would be negative since the SD is often negative. This would match with Figure 7B; 4) Can the graphs that determined the slopes for Figure 7A be shown? Similar to the graph in Figure B?; 5) Please provide the justification for the use of ln(x) for Figure 7B? We thank the reviewer for these helpful suggestions. We agree, and we have made major revisions and edits accordingly to both the original Figure 7 and figure legend, which are included as the new Figure 9. The revisions/edits include updates of labeling of axes and use of a linear correlation instead of the ln(x) display. Please see also our responses provided above to Major points 1 and 2 on the new primary metric outcome measure and use of SD to allow for comparisons between samples.

Please, should not use the word "slope" to describe the "regression coefficient" because that implies a straight line. As in the slope of a tangent line as a certain point on the graph.

Please see response to the previous comment above (1st Minor point). We have updated the original Figure 7 to a new Figure 9, which is now using a linear correlation display.

Would also be helpful to see the Figure 7B graph shown with ln(x) on the x-axis, so we can see how the log transformation made the data look more linear.

Please see our response above to the 1st minor point for this comment as well. We have updated the original Figure 7 to a new Figure 9, which is now using a linear correlation display.

How was the 50 PPN-fibers threshold decided? The threshold for the number of fibers to be included in the determination of the distance to the kth nearest neighbor, i.e. the 50th nearest neighbor in the present study, may be influenced by various factors, including the total number of nerve fibers within the ventral root and the diameter range of the nerve fibers within each sample. To identify a safe threshold for the analyses of each individual ventral root (VR), we used an L-function approach as an established strategy to assess point patterns within a circle of known diameter (Baddeley et al., 2000). We have expanded the revised Figure 2 to include a display of this step in the analyses. In short, the normalized L-function was used to study the general distributions of myelinated fiber centroids of each VR sample and to guide the selection of the optimal kth nearest neighbor used for the analysis. Nerve fiber clustering was detectable for circle spaces with a diameter larger than approximately 20 µm, influenced by the myelinated axon size range with the largest fibers encountered in the L6-S3 VRs being approximately 20 µm in diameter. We next calculated the intercepts of the normalized L-function for each sample VR when calculating the distance for each nerve fiber to the nearest 25th, 50th, and 75th neighboring fiber (Figure 2B). Use of the 50th neighbor for determining possible clustering nerve fiber behavior was selected, as it represented a secure number across all 30 VRs in the present sample.

2) Very last sentence of Results section states "[I]ncreased presence of myelinated PPN-fibers in L6-S3 VRs of rhesus macaques is associated with increased clustering and heterogeneity in the 2D-distributon of nerve fibers." Later in the discussion, this heterogeneity is referred to as heterogeneity in degree of clustering, but here in the results, this can be interpreted as heterogeneity in the fiber types, which would likely need some pie charts with the percentages of each fiber type to be in included in Figure 4.

We agree. In the original text, the term heterogeneity was used with regards to clustering and fiber types in different sentences. We have in the revised version of the manuscript reworded the text and restricted the term heterogeneity to descriptions of clustering. The 2D-distribution analysis of fiber clustering in the present study is based on archival primate ventral root material in the investigator lab and has been used for quantitative analysis of fiber types in lumbosacral ventral roots in a prior study (Bartmeyer et al., 2020). In the revised manuscript, we have included expanded data sets. In the new Figure 7, the 2D distribution of the different fiber types is shown as well as total fiber counts and a histogram display of the percentages for α-MN, γ-MN, and PPN fibers for all individual ventral roots.

3) In the results section "...(PPN-fibers),...(γMN-fibers), and...(α-MN-fibers), respectively [21]" has a different citation style than the rest of the paper, which also cannot be fixed by editors since "21" does not match with the reference style given.

We thank the reviewer for bringing this to our attention. We have corrected the citation to match the citation style for the rest of the manuscript.

4) Use of "2-D" in title and "2D" in rest of the paper. Please correct for consistency.

Thank you. We agree. Done.

5) Clarity needed:"[A]utonomic nerve fibers may extend in the presence of varied number of somatic motor and sensory fibers in nerve roots and peripheral nerves." The word "extend" is unclear. Do the authors mean that the presence of other fiber types/calibers affects the excitation of autonomic fibers? We have reworded the sentence to clarify that autonomic fibers may be found in the peripheral nervous system in the company of somatic motor and sensory fibers in both nerve roots and peripheral nerves.

6) In the discussion, the sentence "For instance, PPN-fibers originate primarily within the segments of the rat spinal cord" seems incomplete. (which segments??) Next sentence on cats describes specifically "2-3 consecutive sacral segments" and next on humans specifically denotes "S2-S5".

We thank the reviewer for bringing this to our attention. We have corrected and completed the sentence that describes the anatomical information on segmental and nerve root origin for PPN-fibers in different species.

Reviewer 2 - In this manuscript the authors present a method for D2 distribution analyses (i.e. grouping) of nerve fibers. The rationale for pursuing this study is justified and relevant, while rather specialized. The methodology was tested and developed using ventral roots of 6 non-human primates, which specimens were collected in the past as part of root avulsion studies. The tissues were collected and processed carefully, and the heterogeneity in nerve fiber composition of these VRs puts the methodology to the test. As for the statistical modeling and interpretation of the modeling results, several queries came up:

1) The clustering model presented is based upon inter-point distances of fibers and number of fibers: isn't this clustering biased towards smaller fibers, simply as larger numbers of smaller fibers can be found closer to each other? Is that why we are seeing the clearest clustering in the panels with higher abundance of PPNs (Figure 6). Is this also why only clusters of tiny fibers remain when the distance is adjusted further (-10) as in Figure 5? Is this the reason that the methodology doesn't find as many clusters when the VRs are mainly composed of large profiles (as in the VRs with mainly aMNs and gMNs), as thresholds were set for a different "population" of nerve fibers? Multiple factors may influence nerve fiber cluster detection. For instance, both the number of fibers needed to form a cluster and the cluster threshold as expressed as the likelihood for a particular 2D-distribution to be a chance occurrence, expressed in SD units. The use of SD units to normalize for nerve root size and for fiber size allows for comparisons between different nerve fiber types, based on size criteria, and between nerve roots with different cross-sectional areas or total number of fibers. Consequently, we believe the observed difference clustering between PPN, α-MN, and γ-MN fibers represents a biological finding.

2) Figure 1: the VR section is discontinuous due to vascular space and the 6x6 squares appear to capture these "holes". A grid from which the "holes, i.e. vascular space" are subtracted, similar to exclusion of empty space outside the VR, would provide a more robust representation.

While Figure 1 still serves it's purpose within the context of the narrative, the same "holes" carry forward in the modeling part as shown in Figure 2. To what extent do these "holes" color or skew the findings of the modeling study? This is especially relevant as the vascular spaces may vary among levels and monkeys.

We thank the reviewer for this comment. We agree that by carrying forward the "holes", which represent vascular spaces into the modeling part of the study, the results may have skewed the findings. In the revised manuscript, we have identified and removed vascular spaces from the endoneurium space used for nerve fiber analysis and modeling. All quantitative data and figures throughout the manuscript have been updated accordingly.

Additional comments:

Figure 2: What do the unlabeled circles in the brown and magenta VRs represent? In our revised Figure 2, the unlabeled circle in the magenta display of ground truth distribution of myelinated nerve fibers in an S1 VR shows an example of the shortened distance (radius/diameter) for enclosing the 50th closest neighbors for a myelinated nerve fiber included in a cluster. The figure legend has been expanded for clarification.

Figure 2C: As the data from all 5 VRs was obtained from the same monkeys, it would be interesting to see how the data from individual monkeys behaves (connect the dots between levels). Thank you. We agree and have added new Figures 6 and 7 to show the data from all L6-S3 VRs in all animals with regards to, for instance, VR size and shape, number of myelinated nerve fibers, 2D distribution of different functional types of nerve fibers, and clustering.

Figure 6: It would be nice to see a similar panel of the raw images and the distribution of the aMNs, gMNs, and PPNs, to fully assess how the modeling behaves in relation to the "true" data.

We have added additional ground truth data to the Results section, including a new Figure 6 inclusion of the raw images of the toluidine blue-stained semi-thin sections of plastic-resin embedded L6-S3 ventral roots for all six animals. We have also a new Figure 7 with the 2D-distribution of segmented myelinated fibers displayed as separate populations of PPNs, α-MN, and γ-MN.
