# Supplementary material for: Nonbinary 2D Distribution Tool Maps Autonomic Nerve Fiber Clustering in Lumbosacral Ventral Roots of Rhesus Macaques
Source: eNeuro. 2024 Apr 11;11(4):ENEURO.0009-23.2024. doi: 10.1523/ENEURO.0009-23.2024 (PMC11015947; doi:10.1523/ENEURO.0009-23.2024)
Supplement: Extended Data 1. — Code for 2-D distribution analysis. Download Extended Data 1, DOCX file. [file eneuro-11-ENEURO.0009-23.2024-s001.docx]

**Extended Data 1**. Code for 2-D distribution analysis

rm(list = ls()) # clear the R environment

library(spatstat)

library(data.table)

library(dbscan)

library(manipulate)

library(latex2exp)

min_int = 4 # maximum diameter for a small fiber

med_int = 10 # maximum diameter for a medium fiber

lim_size <- 10 # number of neighbors to be considered in the d_ik analysis

path <- "~/Dropbox/[xxxxxxxxxx/SpatialDistribution/." # path for the folder with the dataset

setwd(path)

v <- list("Monkey_1") # name of the instance to be analyzed

for(instance in v){

##############################################

newfolder <- "Results" # name of the folder were the results will be stored

new_path <- file.path(dirname(path), paste("Data/",instance,"/",newfolder, sep=""))

# create a folder called "Results" where the images and .csv files will be stored

if (!dir.exists(new_path)){

dir.create(new_path)

}

###########################################################################################

############################# Reading data

###########################################################################################

xdata <- fread(sprintf("Data/%s/data.txt", instance),select = c(1)) # read the x coordinate of each point (first column of the spreadsheet c(1))

ydata <- fread(sprintf("Data/%s/data.txt", instance),select = c(2)) # read the y coordinate of each position (second column of the spreadsheet c(2))

zdata <- fread(sprintf("Data/%s/data.txt", instance),select = c(3)) # read the diameter size of each fiber (third column of the spreadsheet c(3))

# scale the x and y coordinates using z-score

x <- scale(as.numeric(unlist(xdata)))

y <- scale(as.numeric(unlist(ydata)))

z <- as.numeric(unlist(zdata))

#################Plot Diameter histogram

pdf(sprintf('Data/%s/%s/histogram_diameter_%s.pdf', instance, newfolder, instance))

hist(z, breaks = 20, col = "darkblue", main= NULL, xlab = "Diameter (um)")

dev.off()

###########################################################################################

############################# Converting diameter to groups

###########################################################################################

# the fibers will be divided by diameter size considering the values set on line 11 and 12

# if using a dataset where the groups are already defined, one can read the information from the .csv and skip this step

size_small <- 0

size_medium <- 0

size_large <- 0

zdata_aux <- c()

lim_size <- 10

small_aux <- c()

medium_aux <- c()

large_aux <- c()

xy <- cbind(xdata,ydata)

sum_small <- 0

sum_medium <-0

sum_large <- 0

matrix_dist <- as.matrix(dist(xy))

for (i in 1:length(z)){

if (z[i]<= min_int){

zdata_aux[i] <- "small"

size_small <- size_small +1

aux_dist <- sort(matrix_dist[,i],partial=lim_size)

sum_small<- sum_small + aux_dist[lim_size]

}else{

if (z[i]> med_int){

zdata_aux[i] <- "large"

size_large <-size_large +1

aux_dist <- sort(matrix_dist[,i],partial=lim_size)

sum_large <- sum_large + aux_dist[lim_size]

}else{

zdata_aux[i] <- "medium"

size_medium <- size_medium +1

aux_dist <- sort(matrix_dist[,i],partial=lim_size)

sum_medium <- sum_medium + aux_dist[lim_size]

}

}

}

xyz <- cbind(x,y,z)

########## Save the number of fiber in each group in a csv file

cat(size_small, file = sprintf('Data/%s/%s/tamanho_amostra_marks_%s.csv', instance, newfolder, instance), sep = "\n") # Apply cat & append

cat(size_medium, file = sprintf('Data/%s/%s/tamanho_amostra_marks_%s.csv', instance, newfolder, instance), sep = "\n", append = TRUE)

cat(size_large, file = sprintf('Data/%s/%s/tamanho_amostra_marks_%s.csv', instance, newfolder, instance), sep = "\n", append = TRUE)

########## Create a barplot with the percentage of each type of fiber

size_all <- size_small + size_medium + size_large

aux_print <- sprintf("n=%d",size_all)

plot_all <- c(100*size_small/size_all, 100*size_medium/size_all,100*size_large/size_all)

pdf(sprintf('Data/%s/%s/barplot_frequency_%s.pdf', instance, newfolder, instance))

barplot(plot_all, col = c("darkseagreen4", "darkorchid4", "lightsalmon2"), cex.lab=2,cex.axis=2, las = 1, yaxp = c(0, 100, 2), ylim = c(0,100),space=c(0.0,0.1,0.1), ylab = "Percentage")

text(2, 85, aux_print, cex=4, pos=3,col="black") # print the total number of fibers on the graph

dev.off()

############################################################

########################## Cluster Analysis

############################################################

teste_xy <- ppp(x,y, c(min(x)-1,max(x)+1), c(min(y)-1,max(y)+1))

ch<-convexhull(teste_xy) # create the convex hull generated by the points

teste_xy <- ppp(x,y,window = ch,marks = as.factor(zdata_aux)) # incorporate the convex hull to the point pattern

################ Quadrat counting

Q <- quadratcount(teste_xy, nx = 8, ny =8) # count the number of centroids in each square. The reagion will divided in 8X8 squares

pdf(sprintf('Data/%s/%s/quadrat_counting_%s.pdf', instance, newfolder, instance))

plot(intensity(Q, image=TRUE), main=NULL, las=1) # Plot density raster

dev.off()

########## density plot

den <- density(teste_xy, kernel = "disc") # create a density function to represent the centroid distribution

pdf(sprintf('Data/%s/%s/density_%s.pdf', instance, newfolder, instance))

plot(den, main=NULL, las=1)

contour(den, add=TRUE)

dev.off()

############################################################

########################## Group analysis

############################################################

######## split position

split_teste_xy <- split(teste_xy) # devide the dataset by groups

pdf(sprintf('Data/%s/%s/split_%s.pdf', instance, newfolder, instance))

plot(split_teste_xy$large, main=NULL, cex = 0.5,pch = 19, cols = "blue")

points(split_teste_xy$medium, cex = 0.5,pch = 19, col = "green")

points(split_teste_xy$small, cex = 0.5,pch = 19, col = "red")

dev.off()

df <- data.frame(x,y,zdata_aux)

small_teste <- subset(df, zdata_aux == "small")

medium_teste <- subset(df, zdata_aux == "medium")

large_teste <- subset(df, zdata_aux == "large")

######## remove duplicate entries

small_teste <- unique(small_teste[,1:2])

medium_teste <- unique(medium_teste[,1:2])

large_teste <- unique(large_teste[,1:2])

###########################################################################################

############################# Test normal dispersion

###########################################################################################

########## distance of the 10 nearest neighbors

if (size_small > lim_size){

ann.small <- sum_small/size_small

}else{

ann.small <- 0

}

if (size_medium > lim_size){

ann.medium <-sum_medium/size_medium

}else{

ann.medium <-0

}

ann.large <- sum_large/size_large

n <- 1000 # Number of simulations

ann.r <- vector(length = n) # Create an empty object to be used to store simulated ANN values

for (i in 1:n){

rand.p <- rpoint(n=n, win=ch) # Generate random point locations within the convex hull

ann.r[i] <- mean(nndist(rand.p, k= lim_size)) # Tally the ANN values

}

# update the ann.r using the standard deviation

ann.rbar <- vector(length = n) # Create an empty object to be used to store simulated ANN values

for (i in 1:n){

ann.rbar[i] <- (ann.r[i]-mean(ann.r))/sd(ann.r) # Tally the ANN values

}

if(size_small >1){

ann.smallbar <- (ann.small-mean(ann.r))/sd(ann.r)

}else{

ann.smallbar <- 0

}

ann.mediumbar <- (ann.medium-mean(ann.r))/sd(ann.r)

ann.largebar <- (ann.large-mean(ann.r))/sd(ann.r)

############### save figures using standard deviation distance

pdf(sprintf('Data/%s/%s/ANN_%s.pdf', instance, newfolder, instance))

hist(ann.rbar, main=NULL, las=1, breaks=10, col="bisque",

xlim=range(min(ann.smallbar,ann.mediumbar,ann.largebar, ann.rbar), max(ann.smallbar,ann.mediumbar,ann.largebar, ann.rbar)), xlab = TeX('$S_{UG}$'))

#if(size_small > 10){

abline(v=ann.smallbar, col="blue", lwd = 3)

#}

abline(v=ann.mediumbar, col="green", lwd = 3)

abline(v=ann.largebar, col="blue", lwd = 3)

legend(x = "top", legend=c('Small', 'Medium', 'Large'), pch=c(19, 19), col=c('red', 'green', 'blue')) # plot legend

dev.off()

############### save figures using original distance

pdf(sprintf('Data/%s/%s/ANN_rawdistance_%s.pdf', instance, newfolder, instance))

hist(ann.r, main=NULL, las=1, breaks=20, col="bisque",

xlim=range(min(ann.small,ann.medium,ann.large, ann.r), max(ann.small,ann.medium,ann.large, ann.r)), xlab = TeX('$D_{UG} (um)$'))

#if(size_small > 10){

abline(v=ann.small, col="red", lwd = 3)

#}

abline(v=ann.medium, col="green", lwd = 3)

abline(v=ann.large, col="blue", lwd = 3)

legend(x = "top", legend=c('Small', 'Medium', 'Large'), pch=c(19, 19), col=c('red', 'green', 'blue')) # plot legend

dev.off()

############### save an image of one of the simulations generated in the previous step

pdf(sprintf('Data/%s/%s/simulated_pattern_%s.pdf', instance, newfolder, instance))

plot(rand.p, cols = "bisque", pch =19, cex =.5, main = NULL)

dev.off()

###########################################################################################

############################# Test small

###########################################################################################

cluster_mark <- c()

cluster_sd <- c()

x_cluster <- c()

y_cluster <- c()

dist_center_small <- c()

if(nrow(small_teste)> 0.01*length(x) && nrow(small_teste)>lim_size ){

a<- kNNdist(small_teste, k= 1)# max(lim_size,0.01*length(x)))

res <- dbscan(small_teste, eps = mean(a), minPts = max(lim_size,0.01*length(x)))

res <- optics(small_teste, minPts = max(lim_size, 0.01*length(x)))

res <- extractDBSCAN(res, eps_cl = mean(res$coredist))

clusters <- res$cluster

n_cluster <- max(res$cluster)

for (i in 1:n_cluster){

aux <- cbind(small_teste,clusters)

aux_cluster <- subset(aux, clusters == i)

if (nrow(aux_cluster) > lim_size){

aux_dist <- kNNdist(aux_cluster[,1:2], k=lim_size)

ann.aux <- (mean(aux_dist)-mean(ann.r))/sd(ann.r)

saida <- ks.test(a, aux_dist)

if (saida$p.value >= 0.0){

cluster_mark <- cbind(cluster_mark, i)

cluster_sd <- cbind(cluster_sd, ann.aux)

x_cluster <- cbind(x_cluster, mean(aux_cluster[,1]))

y_cluster <- cbind(y_cluster, mean(aux_cluster[,2]))

}

dist_center_small <- cbind(dist_center_small, sqrt((mean(x)-x_cluster[i])^2+(mean(y)-y_cluster[i])^2))

}

}

centers_small <- rbind(x_cluster,y_cluster)

write.table(centers_small, sep = "\t", file = sprintf('Data/%s/%s/cluster_center_small_%s.csv', instance, newfolder, instance))

#hullplot(small_teste, res)

aux_aux <- clusters %in% cluster_mark

clusters[aux_aux == FALSE]<- 0

pdf(sprintf('Data/%s/%s/small_cluster_optics_%s.pdf', instance, newfolder, instance))

plot(small_teste, col = clusters + 1L, pch = clusters + 1L, xaxt="n",yaxt="n",ylab="",xlab="")

dev.off()

pdf(sprintf('Data/%s/%s/small_reachability_optics_%s.pdf', instance, newfolder, instance))

plot(res)

dev.off()

}else{centers_small <- c() }

###########################################################################################

############################# Test medium

###########################################################################################

x_cluster <- c()

y_cluster <- c()

#n_cluster <- max(res$cluster)

clusters <- res$cluster

cluster_mark <- c()

cluster_sd<- c()

dist_center_medium <- c()

centers_medium <- c()

if(nrow(medium_teste) > 0.01*length(x) && nrow(medium_teste) > lim_size){

a<- kNNdist(medium_teste, k= 1)#max(lim_size,0.01*length(x)))

res <- dbscan(medium_teste, eps = mean(a), minPts = max(lim_size,0.01*length(x)))

res <- optics(medium_teste, minPts = max(lim_size,0.01*length(x)))

#dend <- as.dendrogram(res)

res <- extractDBSCAN(res, eps_cl = mean(res$coredist))

clusters <- res$cluster

pdf(sprintf('Data/%s/%s/medium_reachability_optics_%s.pdf', instance, newfolder, instance))

plot(res)

dev.off()

for (i in 1:max(res$cluster)){

aux <- cbind(medium_teste,clusters)

aux_cluster <- subset(medium_teste, clusters == i)

if (nrow(aux_cluster) > lim_size){

aux_dist <- kNNdist(aux_cluster[,1:2], k=lim_size)

ann.aux <- (mean(aux_dist)-mean(ann.r))/sd(ann.r)

saida <- ks.test(a, aux_dist)

if (saida$p.value >= 0.0){

cluster_mark <- cbind(cluster_mark, i)

cluster_sd <- cbind(cluster_sd, ann.aux)

x_cluster <- cbind(x_cluster, mean(aux_cluster[,1]))

y_cluster <- cbind(y_cluster, mean(aux_cluster[,2]))

}

dist_center_medium <- cbind(dist_center_medium, sqrt((mean(x)-x_cluster[i])^2+(mean(y)-y_cluster[i])^2))

}

}

aux_aux <- clusters %in% cluster_mark

clusters[aux_aux == FALSE]<- 0

pdf(sprintf('Data/%s/%s/medium_cluster_optics_%s.pdf', instance, newfolder, instance))

plot(medium_teste, col = clusters + 1L, pch = clusters + 1L, xaxt="n",yaxt="n",ylab="",xlab="")

dev.off()

centers_medium <- rbind(x_cluster,y_cluster)

write.table(centers_medium, sep = "\t", file = sprintf('Data/%s/%s/cluster_center_medium_%s.csv', instance, newfolder, instance))

}else{centers_medium <- c()}

###########################################################################################

############################# Test large

###########################################################################################

x_cluster <- c()

y_cluster <- c()

#n_cluster <- max(res$cluster)

#clusters <- res$cluster

cluster_mark <- c()

cluster_sd<- c()

dist_center_large <- c()

if(nrow(large_teste) >= 0.01*length(x) && nrow(large_teste)>10){

a<- kNNdist(large_teste, k= 1)#max(lim_size,0.01*length(x)))

res <- dbscan(large_teste, eps = mean(a), minPts = max(lim_size,0.01*length(x)))

pdf(sprintf('Data/%s/%s/large_cluster_%s.pdf', instance, newfolder, instance))

plot(large_teste, col = res$cluster + 1L, pch = res$cluster + 1L, xaxt="n",yaxt="n",ylab="",xlab="")

dev.off()

res <- optics(large_teste, minPts = max(lim_size,0.01*length(x)))

res <- extractDBSCAN(res, eps_cl = mean(res$coredist))

clusters <- res$cluster

pdf(sprintf('Data/%s/%s/large_reachability_optics_%s.pdf', instance, newfolder, instance))

plot(res)

dev.off()

for (i in 1:max(res$cluster)){

aux <- cbind(large_teste,clusters)

aux_cluster <- subset(large_teste, clusters == i)

if(nrow(aux_cluster) > lim_size){

aux_dist <- kNNdist(aux_cluster[,1:2], k=lim_size)

ann.aux <- (mean(aux_dist)-mean(ann.r))/sd(ann.r)

saida <- ks.test(a, aux_dist)

if (saida$p.value >= 0.0){

cluster_mark <- cbind(cluster_mark, i)

cluster_sd <- cbind(cluster_sd, ann.aux)

x_cluster <- cbind(x_cluster, mean(aux_cluster[,1]))

y_cluster <- cbind(y_cluster, mean(aux_cluster[,2]))

}

dist_center_large <- cbind(dist_center_large, sqrt((mean(x)-x_cluster[i])^2+(mean(y)-y_cluster[i])^2))

}

}

aux_aux <- clusters %in% cluster_mark

clusters[aux_aux == FALSE]<- 0

pdf(sprintf('Data/%s/%s/large_cluster_optics_%s.pdf', instance, newfolder, instance))

plot(large_teste, col = clusters + 1L, pch = clusters + 1L, xaxt="n",yaxt="n",ylab="",xlab="")

dev.off()

centers_large <- rbind(x_cluster,y_cluster)

write.table(centers_large, sep = "\t", file = sprintf('Data/%s/%s/cluster_center_large_%s.csv', instance, newfolder, instance))

}

lim_size <- 5

df_all <- data.frame(x,y)

#################### save the clusters generated when considering all groups

res <- optics(df_all, minPts = lim_size)

pdf(sprintf('Data/save_all_dbscan2.pdf'))

manipulate(plot(df_all, asp= 1, axes=FALSE, ann = FALSE,col = extractDBSCAN(res, eps_cl = i)$cluster + 1L, pch = extractDBSCAN(res, eps_cl = i)$cluster), i = slider(min(res$coredist),max(res$coredist),step=0.01, initial =mean(res$coredist)*1.25, label = "SD-distance"))

dev.off()

}

################################################################################

#################### Iteractive graph

################################################################################

if(size_small > lim_size){

res <- optics(small_teste, minPts = lim_size)

manipulate(plot(small_teste, asp= 1, axes=FALSE, ann = FALSE,col = extractDBSCAN(res, eps_cl = i)$cluster + 1L, pch = extractDBSCAN(res, eps_cl = i)$cluster), i = slider(min(res$coredist),max(res$coredist),step=0.01, initial =mean(res$coredist), label = "SD-distance"))

}

if(size_medium > lim_size){

res <- optics(medium_teste, minPts = lim_size)

manipulate(plot(medium_teste, asp= 1, axes=FALSE, ann = FALSE,col = extractDBSCAN(res, eps_cl = i)$cluster + 1L, pch = extractDBSCAN(res, eps_cl = i)$cluster), i = slider(min(res$coredist),max(res$coredist),step=0.01, initial =mean(res$coredist), label = "SD-distance"))

}

if(size_large > lim_size){

res <- optics(large_teste, minPts = lim_size)

manipulate(plot(large_teste, asp= 1, axes=FALSE, ann = FALSE,col = extractDBSCAN(res, eps_cl = i)$cluster + 1L, pch = extractDBSCAN(res, eps_cl = i)$cluster), i = slider(min(res$coredist),max(res$coredist),step=0.01, initial =mean(res$coredist), label = "SD-distance"))

}
